# Fundamental Concepts of Hydrogels: Synthesis, Properties, and Their Applications

**DOI:** 10.3390/polym12112702

**Published:** 2020-11-16

**Authors:** Shahid Bashir, Maryam Hina, Javed Iqbal, A. H. Rajpar, M. A. Mujtaba, N. A. Alghamdi, S. Wageh, K. Ramesh, S. Ramesh

**Affiliations:** 1 Centre for Ionics University of Malaya, Department of Physics, Faculty of Science, University of Malaya, Kuala Lumpur 50603, Malaysia; maryamhina@bzu.edu.pk (M.H.); rameshkasi@um.edu.my (K.R.); 2 Center of Nanotechnology, King Abdulaziz University, Jeddah 21589, Saudi Arabia; iqbaljavedch@gmail.com; 3 Mechanical Engineering Department, Jouf University, Sakaka 42421, Saudi Arabia; altafrajpar@yahoo.com; 4 Department of Mechanical Engineering, Center for Energy Science, University of Malaya, Kuala Lumpur 50603, Malaysia; M.mujtaba@uet.edu.pk; 5 Department of Physics, Faculty of Science, Albaha University, Alaqiq 65779-77388, Saudi Arabia; naa.alghamdi@bu.edu.sa; 6 Department of Physics, Faculty of Science, King Abdulaziz University, Jeddah 21589, Saudi Arabia; wageh1@yahoo.com

**Keywords:** hydrogels, hydrogel electrolytes, natural and synthetic polymers, synthesis of hydrogels, properties, applications

## Abstract

In the present review, we focused on the fundamental concepts of hydrogels—classification, the polymers involved, synthesis methods, types of hydrogels, properties, and applications of the hydrogel. Hydrogels can be synthesized from natural polymers, synthetic polymers, polymerizable synthetic monomers, and a combination of natural and synthetic polymers. Synthesis of hydrogels involves physical, chemical, and hybrid bonding. The bonding is formed via different routes, such as solution casting, solution mixing, bulk polymerization, free radical mechanism, radiation method, and interpenetrating network formation. The synthesized hydrogels have significant properties, such as mechanical strength, biocompatibility, biodegradability, swellability, and stimuli sensitivity. These properties are substantial for electrochemical and biomedical applications. Furthermore, this review emphasizes flexible and self-healable hydrogels as electrolytes for energy storage and energy conversion applications. Insufficient adhesiveness (less interfacial interaction) between electrodes and electrolytes and mechanical strength pose serious challenges, such as delamination of the supercapacitors, batteries, and solar cells. Owing to smart and aqueous hydrogels, robust mechanical strength, adhesiveness, stretchability, strain sensitivity, and self-healability are the critical factors that can identify the reliability and robustness of the energy storage and conversion devices. These devices are highly efficient and convenient for smart, light-weight, foldable electronics and modern pollution-free transportation in the current decade.

## 1. Introduction of Hydrogels

The first reported hydrogel can be traced back to 1960, when Wichterle and Lim synthesized poly (2-hydroxethyl methacrylate) (PHEMA) and utilized it in the contact lens industry with the ability of imbibing moisture while asserting its network structure, demonstrating the modern hydrogel [1,2]. Hydrogels, with their peculiar structure of three-dimensional crosslinked polymer meshwork, have the tendency to absorb considerable amounts of water within their interstices and keep bonding it while maintaining the network structure in the swollen state. The demonstration of such phenomena in hydrogels is due to the availability of polar hydrophilic moieties, for example, SO_3_H, OH, NH_2_, COOH, CONH_2_, etc., along the polymer network as branched groups. The tendency of water absorption in hydrogels is due to the swelling character, which is monitored by the hydrophilicity of attached groups, swelling media, and crosslinked bonding strength. Crosslinking controls water absorption as well as helping in maintaining the network structure in the swollen state [3,4,5,6]. The crosslinkers play a prime role for secondary interactions with biological tissues along with the participation of hydrophilic groups for water uptake [7].

Due to the distinctive characteristic properties, such as biodegradability, biocompatibility, hydrophilicity, superabsorbancy, viscoelasticity, softness, and fluffiness, hydrogels play a prime role in biomedical applications. Apart from that, hydrogels also respond to various stimuli, such as temperature, electric field, magnetic field, biological molecules, and ionic strength [8,9]. Many hydrogels have the ability to increase the dwelling period of drugs due to the mucoadhesive and bioadhesive characteristics which promote them as suitable nominees as drug carriers [10].

Recently, hydrogels attained striking attention for energy storage and conversion applications owing to their semi-solid phase and inherent flexibility. Traditional energy storage and conversion devices suffer from heavy weight, mechanical rigidity, and unsustainability. Additionally, there is another issue of device leakage because commercially available devices contain liquid electrolytes. Liquid electrolytes are expensive and harmful to human beings due to their organic nature. Therefore, hydrogel electrolytes have been introduced into these devices. Hydrogel electrolytes are semi-solid, biocompatible, biodegradable, cost-effective, and environmentally friendly. These characteristics, along with the inherent flexibility, are indispensable towards practical applications in energy storage devices, especially supercapacitors [11]. Electrochemical devices using hydrogels become flexible, stretchable, and elastic and can work under stretching, bending, folding, and twisting. These characteristics are seriously devastating over traditional electrochemical devices. Furthermore, hydrogels are self-healable, which is a critical element for wearable and portable electronic devices. Self-healability or damage management ability makes the devices ideal for smart and light-weight electronics [12,13].

Here in this review, fundamental concepts of materials used in hydrogels, such as natural and synthetic polymers, ways of synthesis, and types of hydrogels based on way of crosslinking have been reviewed in detail. In addition, properties of hydrogels, such as biocompatibility, biodegradability, mechanical strength, and swellability, have been illustrated from the basics to the latest concepts with the recent examples of hydrogels. Lastly, hydrogels as electrolytes, their mechanical and self-healing properties, and applications in biomedical and electrochemical devices have been described.

## 2. Classification of Hydrogels

**Figure 1:**
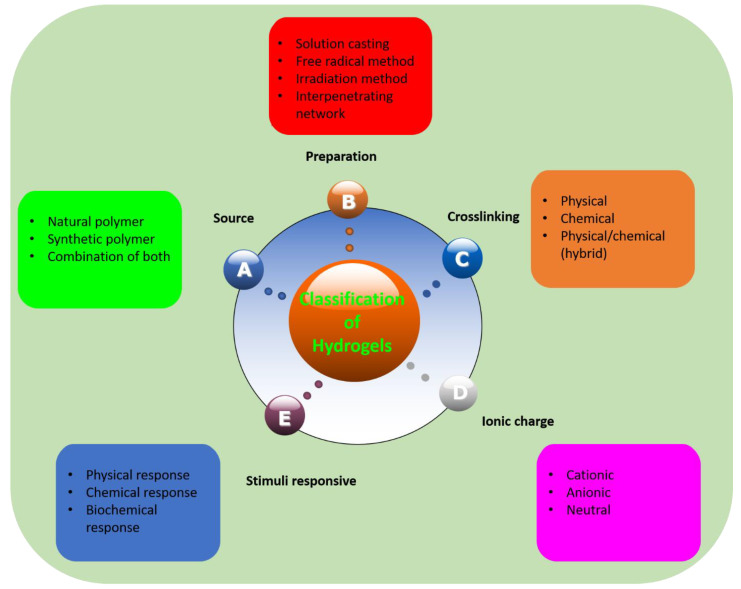
Classification of hydrogels.

Hydrogels can be classified based on different factors and their classification is summed up briefly in Figure 1. Classification of hydrogels depends on the materials (polymers) involved, the source of the polymers, the crosslinking method, their response to stimuli, and their ionic charge. Polymers involved in the hydrogels are natural, synthetic, or combination of natural and synthetic polymers. These polymers can form hydrogels as homopolymer hydrogels, copolymer hydrogels, block copolymer hydrogels, terpolymers, and so on. Moreover, hydrogels are prepared by crosslinking polymers and the crosslinking can be physical, chemical, or both, simultaneously. Crosslinking is formed is numerous ways as well, such as simple mixing, solution casting, bulk polymerization, free radical polymerization, UV and gamma irradiation, and interpenetrating network formation method. Hydrogels can also be classified, based on ionic charge, as cationic, anionic, and neutral hydrogels. The charge on the overall network depends on the charge on the polymer.

## 3. Composition of Hydrogels

Crosslinked polymers are known as hydrogels regardless of whether they are synthetic grafted polymer derivatives or natural and/or a combination of both. Hydrogels comprising of naturally occurring polymers are called natural hydrogels, owing to the characteristic of non-toxicity, and are exuberantly marketed at cheap prices. Natural polymers can be classified into various categories depending upon their chemical structure. These belong to a variety of classes based on their chemical structure: (**i**) polysaccharides (chitin, chitosan, cellulose, starch, gums, alginate, and carrageenan), (**ii**) biological polymers (nucleic acid and DNA), (**iii**) polyamides (collagen), (**iv**) polyphenols (lignin), (**v**) organic polyesters, (**vi**) inorganic polyesters (polyphosphazene), and (**vii**) polyanhydrides (poly sebacic acid) [14]. Hydrogels formed from naturally occurring polymers, especially polysaccharides and proteins, are similar to extracellular matrix due to their natural origin and can easily be identified by the cells and, hence, appear to be biocompatible [15]. Nevertheless, hydrogels synthesized from natural polymers, specifically chitosan and other polysaccharides, are delicate. In order to improve their mechanical properties, natural polymers are crosslinked, grafted with monomers, or blended with synthetic polymers [16]. For example, synthetic polymer poly (vinyl alcohol) is used as a blending agent to increase the mechanical strength and flexibility of natural polymers [17].

Synthetic hydrogels contain synthetic polymers which offer more flexibility to tune the mechanical properties of the hydrogel. The most commonly used synthetic polymers are polycaprolactone [18], poly (vinyl pyrrolidone) (PVP) [19], poly (lactic acid) (PLA) [20], poly (ethylene glycol) (PEG) [21], and poly (vinyl alcohol) (PVA) [22]. PEG is water-soluble, biocompatible, and biodegradable and is especially important as it can conjugate with peptides, proteins, and some other drugs. PEG is also utilized to graft with some natural polymers to induce typical properties required for specific application. Bhattarai et al. synthesized PEG-graft-chitosan as injectable thermo-responsive hydrogels that are liquid at low temperatures but solidify at room temperature [23].

## 4. Natural Polymer Hydrogels

Hydrogels can be categorized as natural, synthetic, or a combination of both. Hydrogels obtained from natural polymers are classified as natural polymer hydrogels. These natural polymers include polysaccharides, polynucleotides, and polypeptides. The natural polymers can be obtained from diverse natural sources and are classified as neutral, cationic, and anionic in nature. These polymers are easy to access, abundant, inexpensive, non-toxic, and biodegradable and possess other attractive biological properties. There has always been a great interest in the relationship of structure and function, especially in natural biologically active compounds. The advances in structural and functional substances over the past few decades made an increasing number of developments in materials to be used in biomedical technology. Natural polymers have well-defined, larger structures formed by covalently bonded monomeric units. Natural polymer hydrogels can be used for numerous biomedical applications, such as controlled and targeted release of drugs, tissue engineering, and wound healing [24,25,26].

### 4.1. Polysaccharide Hydrogels

Polysaccharides are a distinctive class of naturally occurring polymers that acquire a massive variety of structural characteristics. Polysaccharides can be used as renewable biomaterials due to their exceptional biological properties. They are formed from long-chain carbohydrate molecules of repeated monomeric units held by glycosidic bonds. These act as promising biomaterials and carry special physiological functions and biological activities and are helpful for a variety of applications. Naturally available polysaccharides, such as cellulose, starch, chitin, chitosan, carrageenan, alginates, dextran, pullulan, and pectin, are studied extensively for industrial, medical, pharmaceutical, and tissue engineering applications [27].

There has been great interest in the growth and development of polysaccharide hydrogels during the past few years for biomedical applications. Hydrogels loaded with drugs can sustain their levels in blood and can be controlled subcutaneously, orally, and/or intramuscularly [28,29]. Although synthetically-developed biocompatible and biodegradable polymer hydrogels are also useful for biomedical applications, polysaccharides remain smart and attractive due to their abundant availability, non-toxicity, good biocompatibility and biodegradability, ease of modification and preparation, low cost, and renewable physico-chemical properties and, hence, have been used by mankind in different forms [30].

### 4.2. Synthetic Polymer Hydrogels

Synthetic polymers are attractive for the synthesis of synthetic polymer hydrogels as they have highly controllable physical and chemical properties than natural polymers. Synthetic polymers can be produced with long-chain structures and high molecular weight. Unfortunately, synthetic polymer hydrogels have lower biological activity than natural hydrogels. Synthetic polymer hydrogels can be synthesized via numerous ways, employing polymerizable vinyl monomers or chemical crosslinking of polymers. Synthetic polymers used in the synthesis of hydrogels are poly (vinyl alcohol) (PVA), poly (ethylene glycol) (PEG), poly (ethylene oxide) (PEO), poly (2-hydroxyethyl methacrylate) (PHEMA), poly (acrylic acid) (PAA), and poly (acrylamide) (PAAm), etc. Some of synthetic polymer hydrogels are discussed in the sections below.

### 4.3. Hydrogels Based on Poly (Acrylic Acid) Derivatives

Acrylic acid-based hydrogels are pH-sensitive due to the availability of anionic carboxyl groups and show higher swelling at basic pH but low swelling at acidic pH compared to hydrogels containing nonionic or neutral pendant groups. The high swelling at basic pH is owing to the electrostatic repulsion of carboxylate ions formed at basic pH because of deprotonation of carboxylic groups. Acrylic acid-based hydrogels have been extensively prepared and exploited in numerous disciplines, specifically in the biomedical field, due to their excellent adhesive characteristics. Poly (acrylic acid) and glycidyl methacrylate dextran copolymer hydrogels were synthesized using UV irradiation. Synthesized hydrogels demonstrated pH-sensitive swelling in the presence of dextranase at pH 7.4 [31]. Lee et al. reported glycerol crosslinked poly (acrylic acid) hydrogel by polymerizing acrylic acid in the presence of benzoyl peroxide and novozym 435. This hydrogel showed 100% higher swelling in neutral, acidic, and basic pH [32]. Furthermore, poly (acrylic acid) hydrogel was synthesized by free radical co-polymerization using N, N′-methylenebisacrylamide as a crosslinking agent [33]. Nho et al. synthesized poly (acrylic acid) hydrogels by irradiating the acrylic acid solution using an electron beam of maximum 75 kGy, and physical properties, such as swelling ratio, mucoadhesion, and gel contents, were investigated [34].

**Figure 2:**
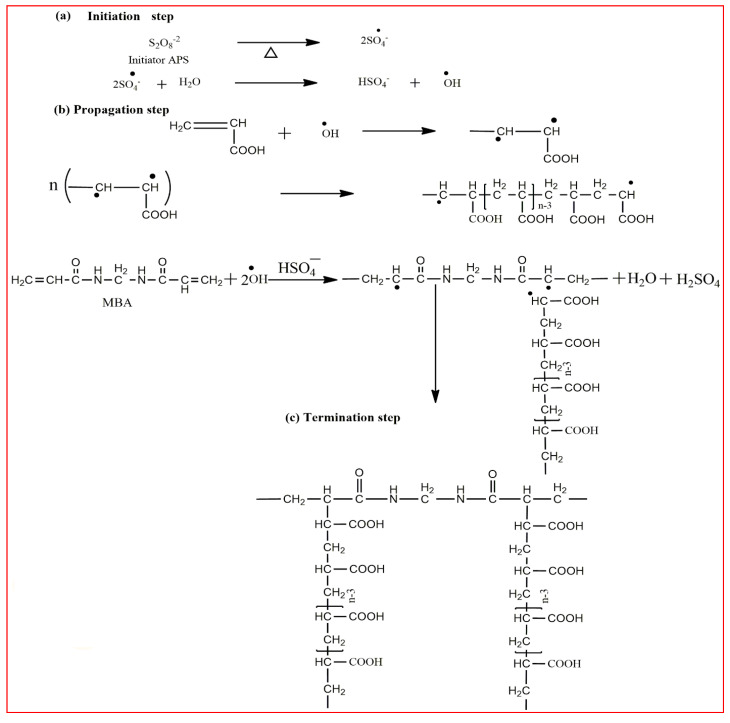
The reaction mechanism of the poly (acrylic acid) hydrogel [38].

Methacrylic acid is a derivative of acrylic acid and it also has significant attraction in the biomedical field. Kou et al. synthesized poly (2-hydroxyethyl methacrylate-co-methacrylic acid) hydrogel slabs and cylinders using free radical polymerization. Hydrogel slabs were synthesized using redox couple ammonium persulfate and sodium metabisulfite as an initiator while using tetraethyleneglycol dimethacrylate (TEGDMA) as a crosslinker. Hydrogel cylinders were synthesized using 2, 2′-azobisisobutyronitrile (AIBN) as an initiator and TEGDMA as a crosslinking agent. Furthermore, phenylpropanolamine drug release was studied at different pH values via a desorption method [35]. In another study, Park et al. synthesized pH-sensitive poly (vinyl alcohol-co-acrylic acid) and poly (vinyl alcohol-co-methacrylic acid) hydrogels via grafting of acrylic acid and methacrylic acid on PVA hydrogels in two steps using gamma irradiation. Initially, PVA hydrogels were synthesized using gamma rays of 50 kGy and then acrylic acid and methacrylic acid were grafted onto PVA hydrogels with an irradiation of 5–20 kGy. Grafted hydrogels exhibit pH-sensitive swelling properties and pH-controlled insulin release behavior [36]. Moreover, an interpenetrating network (IPN) of methacrylic acid and PVA hydrogels was prepared using glutaraldehyde as a crosslinking agent via the water-in-oil emulsion method. The synthesized hydrogels showed a pH-sensitive swelling property and release of ibuprofen [37]. Khan and his research group recently reported a poly (acrylic acid) hydrogel using ammonium persulfate and N, N′-methylenebisacrylamide as an initiator and a crosslinking agent, respectively. The synthesis mechanism of the hydrogel is presented in Figure 2 [38].

### 4.4. Hydrogels Based on Poly (Acrylamide) Derivatives

**Figure 3:**
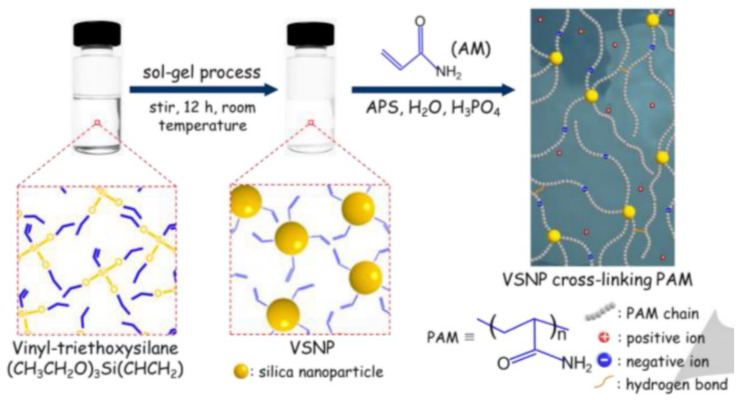
Preparation of vinyl hybrid silica nanoparticles (VSNPs) from vinyl-triethoxysilane nanoparticles followed by the synthesis of VSNP-PAM from VSNPs (crosslinking agent), ammonium persulfate (APS, initiator), acrylamide (AM, main monomer), and phosphoric acid (proton source) [11].

Many acrylamide hydrogels have been prepared and utilized in numerous fields. Poly (acrylamide) hydrogels are hydrophilic, neutral, and possess significant valuable physical and chemical properties for potential applications as a biomaterial, for the immobilization of cells and biocatalysts, in drug delivery systems, for heavy metal ions absorption, and as bio-separators. Saraydin et al. synthesized poly (acrylamide) hydrogels using the chemical free radical initiation and gamma ray irradiation (0.72 kGy hr^−1^) method in the presence of three different crosslinking agents. The synthesized gels displayed different swelling ratios, ranging from 255 to 1450%, depending upon the synthesis protocol, crosslinking agent, and concentration [39]. Poly (acrylamide) hydrogels were also synthesized using vinyl hybrid silica nanoparticles (VSNPs) as a crosslinking agent. Firstly, VSPNs were prepared using vinyl-triethoxysilane via the sol-gel process. The purpose of VSNPs’ introduction was to promote dynamic crosslinking of the poly (acrylamide) network. VSNPs served as crosslinking agents as well as stress buffers to dissipate energy. Preparation of vinyl hybrid silica nanoparticles (VSNPs) from vinyl-triethoxysilane nanoparticles, followed by the synthesis of the VSNP-PAM hydrogel, is demonstrated below in Figure 3 [11].

**Figure 4:**
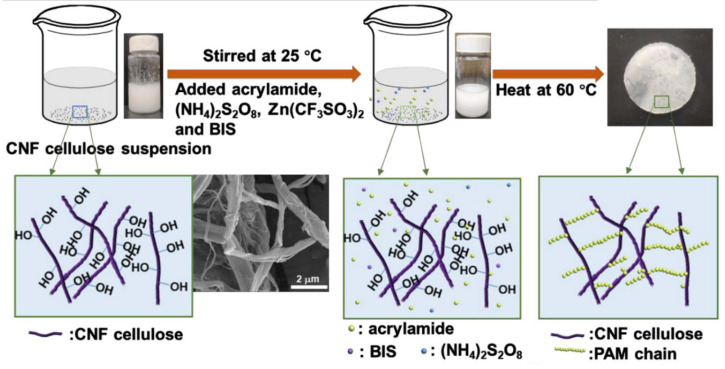
Schematic of the synthesis route to form solid-state electrolytes by grafting PAM on cellulose nanofibers (CNFs) via a facile free radical polymerization approach [40].

Xu et al. reported poly (acrylamide) hydrogel membranes grafted over cellulose nanofibers (CNFs) crosslinked through N, N′-methylenebisacrylamide via free radical mechanism. The grafting was owing to the interconnection of amide group of poly (acrylamide) and hydroxyl and carboxyl groups present on the surface of CNFs (Figure 4) [40].

**Figure 5:**
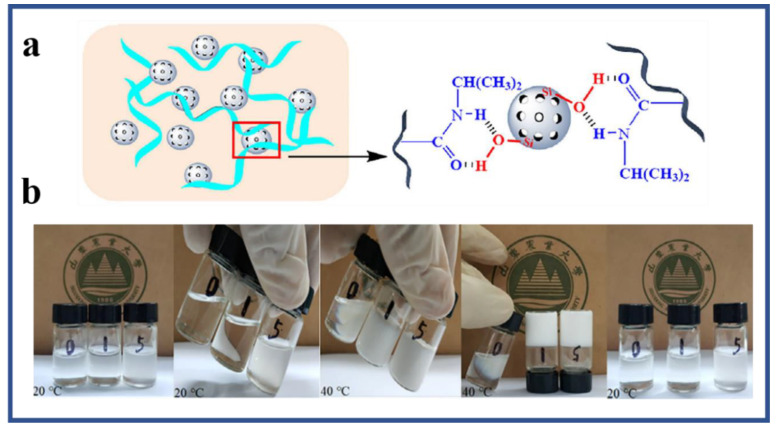
(**a**) Synthesis mechanism of poly (N-isopropylacrylamide)/mesoporous silica nanoparticle (PNIPAM/MSN) composite hydrogels; (**b**) digital photographs showing the PNIPAM/MSN-0, PNIPAM/MSN-1, and PNIPAM/MSN-5 hydrogels at 20 °C and 40 °C [48].

Hina et al. synthesized novel self-healable poly (acrylamide) clay crosslinked hydrogels through the free radical approach [41]. Poly (acrylamide) hydrogels are weak in hydrolytic stability and mechanical strength. Efforts have been made to ameliorate their properties by co-polymerization with other monomers [39]. Poly (acrylamide-co-acrylic acid) hydrogel has been prepared by free radical polymerization. A rheological study of the hydrogels exhibited an increase in the mechanical strength of copolymer hydrogels [42]. In another study, poly (acrylamide-co-acrylic acid)/poly (acrylamide) super porous and IPN hydrogels were prepared by pre-polymerization, synchronous polymerization, and the frothing process. The swelling property was observed to be high while low in compressive strength. Furthermore, the IPN hydrogels showed a decrease in water absorption but an increase in water retention and compression strength [43]. However, poly (alkylacrylamide) hydrogels are thermosensitive, although most hydrogels show negative temperature sensitivity. These hydrogels have a lower critical solution temperature (LCST) and show contraction upon heating above the lower critical solution temperature (LCST) [44]. Poly (N-isopropylacrylamide) (PNIPAM) is the most important thermo-sensitive polymer and its hydrogels have been extensively studied in numerous fields. Matzelle et al. prepared PNIPAM and poly (acrylamide) hydrogels to analyze the effect of temperature on elastic parameters. The elastic properties of the PNIPAM hydrogel were temperature-dependent while the poly (acrylamide) hydrogel had a very slight response to changes in temperature [45]. PNIPAM copolymer hydrogels have been extensively observed as biomaterials for the pulsatile delivery of thrombolytic and antithrombotic streptokinase and heparin [46]. Fathi et al. prepared dual thermo- and pH-sensitive hydrogels of poly (N-isopropylacrylamide-co-itaconic acid) and chitosan. The hydrogels were engineered by blending poly (N-isopropylacrylamide-co-itaconic acid) with chitosan and glycerophosphate was added as an ionic crosslinking agent [47]. Temperature-sensitive hydrogels of poly (N-isopropylacrylamide) are significant in drug delivery and the method of crosslinking plays a role in defining the characteristics of hydrogels. Mesoporous silica nanoparticles (MSNs) improve the network, morphology, and pore size, and drug-loading/release properties. The synthesis mechanism of poly (N-isopropylacrylamide) (PNIPAM) hydrogel using mesoporous silica nanoparticles is presented in Figure 5 [48].

Panayiotou et al. prepared poly (N, N′-diethyl acrylamide) (PNDEAM) and poly (N-isopropylacrylamide) hydrogels through free radical polymerization and compared their swelling–deswelling–reswelling, insulin storage, and controlled release ability. The results revealed the stronger dependency of swelling of the PNDEAM hydrogel on the crosslinking agent, slower reswelling kinetics, and faster insulin release compared to the PNIPAM hydrogel [49].

### 4.5. Natural Polymers in Combination with Synthetic Polymer Hydrogels

Naturally occurring polymers exhibit a weak mechanical strength; therefore, due to this drawback, their applications are limited in biomedical areas. However, synthetic polymers take precedence in this challenge over the naturally occurring ones and are being exploited in various applications due to their facile synthesis protocol, cost effectiveness, and tailor-made properties, suitable for particular application. In addition to that, synthetic polymers show excellent mechanical strength [50,51]. Nevertheless, synthetic polymers are not environmentally friendly because the produced solid waste material could not be degrading biologically.

In chitosan-based hydrogel formulations, formaldehyde is used as a crosslinking agent which releases the drug at a pH equal to the pH of stomach [52]. Chen and his co-workers prepared semi-interpenetrating networks (semi-IPNs) of chitosan-graft-poly (methacrylic acid) via free radical mechanism using formaldehyde as a crosslinker and 2, 2′-azo bis-isobutyronitrile as an initiator [53]. Chitosan-graft-poly (acrylic acid-co-hydroxyethyl methacrylate) hydrogel membranes were synthesized using cerium ammonium nitrate chemical initiator. The grafting of copolymers on chitosan was further characterized using different techniques [54]. Agnihotri et al. fabricated semi-IPN chitosan-poly (acrylamide-co-ethylene oxide) hydrogel microspheres. In the first step, poly (acrylamide-co-ethylene oxide) was prepared by free radical mechanism and embedded in the glutaraldehyde (GA) crosslinked chitosan hydrogel. These hydrogel microspheres were used to encapsulate and deliver anti-cancer drugs [55]. On the other hand, a novel pH-sensitive IPN of acrylamide-grafted-PVA crosslinked with chitosan using glutaraldehyde was used to deliver antibiotic drugs. The hydrogel demonstrated prolonged release of the drug up to 10 h [56]. Stimuli sensitive chitosan grafted poly (acrylic acid), poly (hydroxy propyl methacrylate), PVA, and gelatin hydrogels were prepared using gamma rays. [57]. Lejardi et al. reported hydrogel blends of chitosan and modified PVA-graft-glycolic acid by mixing chitosan solution with modified PVA under constant agitation. These hydrogels showed enhanced rheological properties [58]. A pH-responsive semi-IPN of N-carboxyethyl chitosan and PHEMA hydrogels were synthesized through photo polymerization. This hydrogel exhibited good mechanical properties and sustained release of 5-fluorouracil [59]. Hydrogel films of pectin-grafted acrylamide crosslinked with glutaraldehyde were also reported elsewhere. This formulation showed better film forming, gelling, and mechanical properties compared to pure pectin [60]. Cellulose-supported synthetic polymerizable monomer hydrogels were prepared using chemical, photo, and gamma ray initiation. Some disadvantages of these techniques are the cost and non-availability of required equipment and difficulties in homopolymerization [61]. Interpenetrating network of guar gum and PVA were reported using glutaraldehyde as a crosslinking agent. These IPN hydrogels were used in drug delivery systems [62]. In another study, grafted copolymers of guar gum and acrylamide hydrogel microspheres were synthesized using GA as a crosslinking agent. These hydrogels were further analyzed and tested as an anti-hypertensive drug delivery [63]. Hydrogel microparticles of hydroxyethyl starch-grafted- PHEMA were synthesized by free radical polymerization and protein release was studied. The results showed that protein release was dependent upon hydrogel network density and the size of the entrapped proteins [64]. Other hydrogel formulations, including alginate, carrageenan, alginic acid, gum arabic, and xanthan gum modified with synthetic polymers as well as synthetic polymerizable monomers through different methods, have been reported. In our work, we synthesized hydrogels comprised of natural and synthetic polymers using different crosslinking agents for anti-cancer, anti-asthma, and anti-inflammatory drug delivery at various pH levels [65].

## 5. Types of Hydrogels

Hydrogels based on the type of crosslinking forces between polymeric chains and hydrogels can be classified into chemical hydrogels, physical hydrogels, and a combination of both by crosslinking simultaneously (hybrid). If the crosslinking among the polymer chains is due to non-covalent forces (secondary forces), such as hydrogen bonding, ionic forces, Van der Waals interactions, polyelectrolyte complexation, stereocomplexation, and hydrophobic forces, the hydrogels formed through these interactions are classified as physical hydrogels. Physical hydrogels show a reversible response to environmental changes because the secondary interactions between the polymer chains are not very strong. They are disordered, fragile, and mechanically weak when exposed to external stimuli. Due to weak interactions, physical hydrogels usually dissolve in organic solvents and water upon heating, whereas the formation of chemical hydrogels, also known as permanent hydrogels, involves covalent bonding between the polymer chains. These hydrogels do not dissolve in the surrounding medium and, hence, do not show a reversible response (sol-gel transition) like physical hydrogels do, due to the presence of strong covalent bonding between the macromolecular chains. Chemical crosslinking occurs by various means, utilizing small molecules (formaldehyde, glutaraldehyde (GA), genipin, diglycidyl ether, and N, N′-methylenebisacrylamide). The mechanism involved in order to stabilize the network through condensation reaction or free radical mechanism is that the small molecules form covalent bonds with the polymers. In the case of condensation reactions, the crosslinker should contain two functional groups normally (GA) [66], but a mono functional group containing a crosslinker (formaldehyde) can also be used [67]. In the free radical mechanism, the crosslinker must contain at least two double bonds to bind with the polymer chain on either side. Chemical crosslinking imparts characteristics such as excellent thermal, mechanical, chemical, and surface properties to the prepared hydrogels as well as being responsible for maintaining the network structure of hydrogels in a fully swollen state [68].

### 5.1. Physical Crosslinked Hydrogels

**Figure 6:**
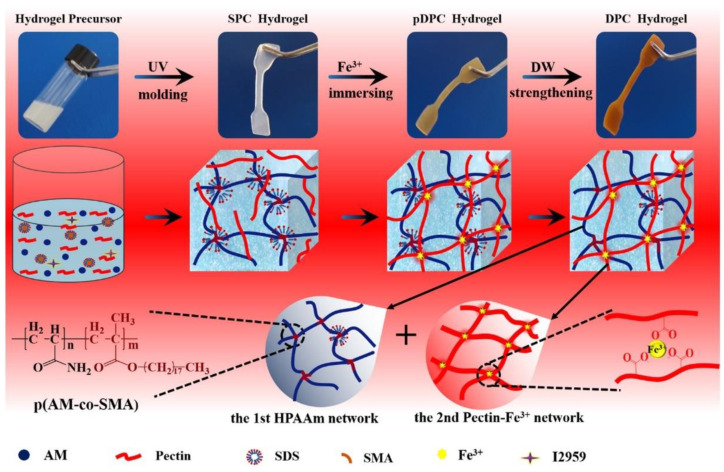
Dual physically crosslinked pectin-Fe^3+^ ion/hydrophobically modified acrylamide hydrogels under ultraviolet light in three steps [74].

Polymer networks should meet the following conditions to form hydrogels: (a) strong inter-chain interactions in order to form a stable colligation in the molecular network, and (b) the polymer network must encourage the access and residence of water within the hydrogel. Hydrogels fulfilling these demands may, perhaps, be prepared by non-covalent approaches, such as electrostatic, hydrogen bonding, and hydrophobic forces among polymer chains. The hydrogels formed by these interactions are solely physical gels and have high water sensitivity and thermo-reversibility [69]. Such a type of hydrogel demonstrates a short lifespan, in the range of a few days to a maximum of a month, in the physiological media. Consequently, physical gels take the advantage and are used where a short-term drug release is required. These types of hydrogels are safe to use for clinical purposes because the gelation does not require any toxic covalent crosslinking molecules. Polysaccharide-based physical hydrogels can be synthesized by mixing the polymers in suitable circumstances. For example, ionic complexation of chitosan with small anionic molecules, i.e., sulfates, phosphates, and citrates or anions of Pt (II), Pd (II), and Mo (VI), can form physical hydrogels. Anions bind to the chitosan via protonated amino group of chitosan. The properties of the formed hydrogels depend on the charge and size of anions as well as the concentration and the level of deacetylation of chitosan. However, chitosan has a slight or no charge above pH 6; this results in a reduction in the ability of chitosan to form ionic complexes and its applications in physiological media [70]. Chitosan derivatives also form physical hydrogels through ionic interaction. N-succinyl chitosan-formed hydrogel beads and blends with alginate in the presence of Ca^2+^ and Zn^2+^ ions [71]. For example, alginate is a well-known polysaccharide that can be crosslinked with Ca^2+^ ions via ionic interaction [72]. Carrageenan comprised of α-D galactose and β-D-galactose with different fractions of sulfate ions can form a hydrogel in the presence of K^+^ ions. Pectin is a heteropolysaccharide extracted from terrestrial plants consisting of α-galacturonic acid residues linked by linear 1, 4-glycosidic linkages. Pectin is also naturally biocompatible and is used to prepare physically crosslinked hydrogels for numerous applications. To enhance the mechanical strength of pectin hydrogels, pectin-Fe^3+^ ion/polyacrylamide hydrogels have been prepared in two steps via a UV method using ab N, N′-methylenebisacrylamide crosslinking agent [73]. Tough pectin hydrogels physically crosslinked with metal ion are characterized by their unique properties and versatile biological functions. Dual physically crosslinked pectin-Fe^3+^ ion/hydrophobically modified acrylamide hydrogels were prepared under an ultraviolet light (365 nm; 8 W) in three steps. The preparation steps are displayed in Figure 6 [74].

Similarly, other polymers, such as sodium alginate and xanthan gum, can interact ionically with Fe^3+^ ions to form hydrogel networks [75,76]. Although one may also form hydrogels in the absence of salt solutions, hydrogels without metal ions are relatively weaker than hydrogels formed in the existence of metal ions [77].

Furthermore, polyelectrolyte hydrogels can also be prepared by complex formation of polycations with polyanions. Chitosan as a polycationic polysaccharide formed complexes with polyanions, such as dextran sulfate or polyphosphoric acid [78]. Other natural-type anionic polysaccharides, such as alginate, carrageenan, carboxymethyl cellulose, pectin, and xanthan, also form complexes with chitosan. Chitosan can form polyelectrolyte complexes with synthetic-type anionic polymers, such as poly (acrylic acid). The major factors which play a prime role in stabilizing these complexes are charge density, pH, solvent, ionic strength, and temperature.

Physical hydrogels can also be prepared by physically melding non-ionic polymers. These polymers form intersection points in the form of crystallites after freeze-dried or freeze-thaw cycles. Hydrogel development is most likely by crystallization due to association of chains via hydrogen bonding. For example, an aqueous solution of PVA formed a gel having weak mechanical strength when kept at ambient conditions. Afterward freeze-thawing, the aqueous solution transformed into PVA hydrogel. The mechanical strength of this hydrogel became stronger than before the process of freeze-thawing. The properties of hydrogels depend on the PVA concentration and the time and number of freeze-thawing cycles [79]. Moreover, the addition of polysaccharides, such as chitosan, reduced the crystallinity of PVA hydrogels, dextran increased the process of crystallization of PVA hydrogels, and alginate increased the mechanical strength of the PVA hydrogels [80,81].

### 5.2. Chemically Crosslinked Hydrogels

The formation of physical gels through clustering of molecules causes formation of free chain loops and thus inhomogeneity that signifies short-lived network imperfections. Chemical crosslinked hydrogel networks are easy to control as compared to physical hydrogels because their synthesis and applications are not only dependent on pH. Chemical crosslinking can be used to transform the physical properties of the hydrogels. Usually, swelling behavior, biodegradability, and mechanical strength have been modulated via covalent crosslinking. Covalent crosslinking can be done through numerous approaches. These are discussed below.

#### 5.2.1. Crosslinking by Small Molecules

Polymers can be crosslinked using mono- and bi-functional small molecules, such as formaldehyde, glutaraldehyde, genipin, diethyl squarate, ethyleneglycol diglycidyl ether (EGDE), and blocked diisocyanate, as revealed in Table 1. Crosslinking of polymers using these small molecules has been reported and reviewed extensively. Covalent crosslinked polymers have an improved mechanical strength compared to physically crosslinked polymers. In spite of offering many attractive properties, the biocompatibility of some crosslinkers is still unknown. In order to avoid the introduction of crosslinking agents, polymers have been functionalized with desired reactive functional groups to synthesize hydrogels in situ. Numerous hydrogels have been prepared based on selective functional groups, as shown in Table 2. Chitosan hydrogels have been synthesized through Schiff bases with other pre-functionalized polysaccharides, such as oxidized dextran and aldehyde hyaluronic acid [82]. Tan et al. prepared N-succinyl chitosan/aldehyde functionalized hyaluronic acid injectable composite hydrogels through a Schiff base mechanism. It was observed that the compressive modulus, which is an important factor for cartilage tissue engineering, improved with an increasing amount of N-succinyl chitosan in the hybrid hydrogel [83]. Similarly, other hydrogels of cellulose and alginate have also been prepared [84,85]. Furthermore, amino groups containing polymers, such as chitosan, and their derivatives form hydrogels via Michael addition reactions. The amino groups react with the vinyl group of other polymers. Metters et al. prepared chitosan-polyethylene oxide (PEO) hydrogels through Michael addition reactions. In the first step, chitosan was treated with 2-carboxyethyl acrylate to form acrylated chitosan and then processed with thiolated PEO through the Michael addition reaction. This method is very popular due to the short reaction time and comparatively compassionate reactivity towards biomolecules. Hydrogels prepared through this method also have enhanced mucoadhesive properties. In spite of many advantages of this method, several disadvantages also exist. Hydrogel preparation using this method involves multi-step preparation and purification. Moreover, polymers might become cytotoxic after functionalization with the reactive groups.

#### 5.2.2. Crosslinking through Ionizing Radiation

**Figure 7:**
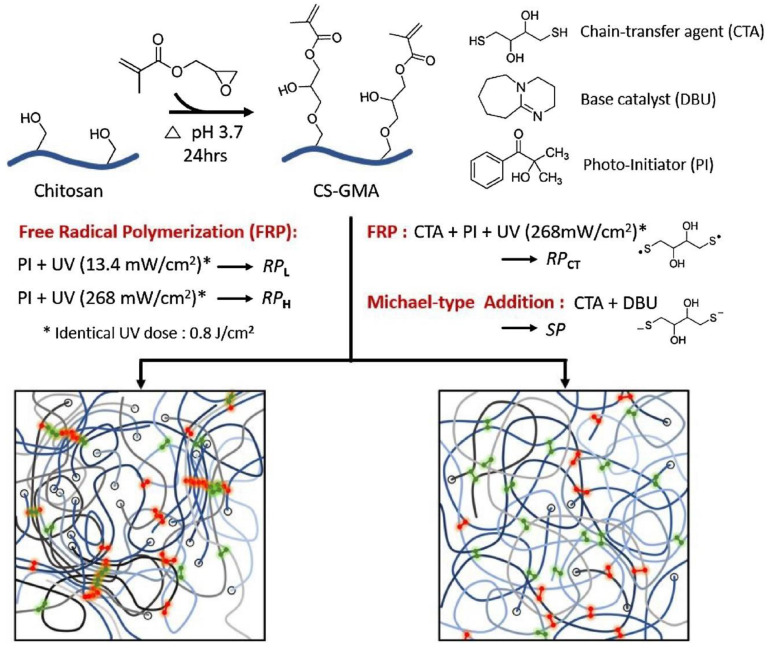
Synthetic routes for chitosan hydrogel preparation and schematic illustrations of the expected network structures of RP_L_ (radical polymerization at low UV intensity) and RP_H_ (**left**) radical polymerization at high UV intensity) or RP_CT_ (chain-transfer radical polymerization) and SP (step polymerization) (**right**). Open circles represent the point at which the chain moves out of the plane. Red and green linkages indicate inter- and intra-chain crosslinking, respectively [88].

Hydrogels have been prepared through light-sensitive functional groups. This technique has significant advantages, such as ease, speedy preparation, and low cost of production compared to chemical crosslinking methods. Ono et al. reported UV-light-irradiated chitosan hydrogels by introducing azide and lactose as light-sensitive moieties. Azide group converted into nitrene group after UV irradiation, which bound to amino groups of chitosan to form a hydrogel in a short time [86]. Other UV-irradiated chitosan hydrogels were prepared by pre-functionalization with photo-sensitive acrylates of chitosan and pluronic acid [87]. Recently, methacrylate-functionalized chitosan hydrogels have been reported using a UV light by step growth or chain-growth polymerization using dithiothreitol (DTT). Furthermore, 2-hydroxy-2-methylpropiophenone and UV LED (365 nm) changing power density, 13.4 or 268 mW/cm^2^, were used as the photo initiator and source of radiation, respectively. Chitosan hydrogels formed by photo-initiated free radical polymerization (FRP) were designated as RP, and the subscripts L or H were used to indicate low (13.4 mW/cm^2^, 60 s) or high (268 mW/cm^2^, 3 s) UV intensity used to initiate FRP. The subscript CT indicated the use of the chain-transfer agent dithiothreitol (DTT) during FRP. Chitosan hydrogels formed by step polymerization via a base-catalyzed thiol-ene reaction were termed SP. The hydrogel synthesis method is shown in Figure 7 [88].

Light-sensitive polymers form hydrogels in situ. Hydrogel formation via this method has some serious drawbacks. Irradiation crosslinking requires a light sensitizer and delayed irradiation, which causes an increase in local temperature and damage of cells and tissues. The functional groups and proposed mechanism of UV light sensitivity are shown in Table 3.

#### 5.2.3. Crosslinking through Free Radical Mechanism

The free radical polymerization of low molecular weight monomers in the presence of crosslinking agent results in the development of chemically crosslinked hydrogels. The initiation reactions start by using an initiator, such as potassium persulfate (KPS), ammonium persulfate (APS), ceric ammonium nitrate, ferrous ammonium sulfate, 2-2′-azobisisobutyronitrile (AIBN), and benzoyl peroxide. In this mechanism, vinyl monomers radically polymerize in the presence of a crosslinker to form chemically crosslinked hydrogels. Vinyl monomers include acrylic acid, acrylamide, vinyl chloride, styrene, epoxide, N-vinyl-2-pyrrolidone, and 2-hydroxyethyl methacrylate. The crosslinking agents used in the free radical mechanism are N, N′-methylenebisacrylamide (MBA), ethyleneglycol dimethacrylate (EGDMA), melamine trimethylacrylamide, and melamine triacrylamide. Crosslinked homopolymers, such as poly (2-hydroxyethyl methacrylate) (PHEMA) and polyvinylpyrrolidone (PVP), and copolymers of N-vinyl-2-pyrrolidone and 2-hydroxyethyl methacrylate (HEMA) are synthesized by free radical polymerization using melamine trimethylacrylamide and melamine triacrylamide as crosslinkers and AIBN as an initiator.

Moreover, free radical mechanism is used to graft synthetic monomers over natural polysaccharides. Grafting is a general way to improve the properties of polymers, such as complexation-, chelating-, and absorption-enhancing properties, and solubility in water as well as in organic solvents. Grafting also improves some interesting properties related to biomedical applications, such as biodegradability, biocompatibility, mucoadhesivity, and mechanical strength. Numerous hydrogels have been reported in the literature, in which natural polymers, such as chitosan, cellulose, starch, pectin, alginate, hyaluronic acid, dextran, carrageenan, and gums, have been grafted by synthetic monomers in the presence of a crosslinking agent to improve their intrinsic properties. Hydrogels of chitosan and its derivatives have been prepared by grafting synthetic monomers, and their properties are extensively investigated. pH-sensitive chitosan-graft-poly (acrylamide) hydrogels have been prepared by Pourjavadi et al. using APS and MBA as an initiator and crosslinker, respectively. The results revealed that swelling ratio is dependent on the acrylamide contents; MBA and alkaline hydrolysis of hydrogels further increased the swelling ratio. Swelling kinetics were observed to be second order kinetics [89]. In another study, Mahdivinia et al. prepared chitosan-graft- poly (acrylamide-co-acrylic acid) hydrogels using KPS as an initiator and MBA as a crosslinker; they studied the effect of pH and salt solution on the swelling properties of hydrogels. Alkaline hydrolysis was also done to observe the swelling properties and it was found that alkaline hydrolyzed hydrogels having a greater amount of acrylamide showed higher swelling ratios and non-hydrolyzed hydrogels having a greater amount of acrylic acid showed greater swelling. This study was in good agreement with a previous one [90]. Moreover, the free radical mechanism is used to prepare carboxymethyl chitosan/acrylic acid hydrogels by copolymerization in the presence of vinyltriethoxysilane as a crosslinker using KPS as an initiator [91]. Pourjavadi et al. synthesized super porous kappa-carrageenan-g-poly (acrylic acid) in air via free radical initiator, APS, in the presence of MBA. The synthesis of the hydrogel was optimized by varying the composition of reaction. Appreciable swelling capacity was reported in different salt solutions, especially in sodium chloride solution due to the anti-salt characteristics of the sulfate groups in carrageenan of the super absorbing hydrogels. Moreover, the results revealed that the presence of air had no significant effect on hydrogels and swelling behavior [92]. The copolymerization and crosslinking of acrylic acid and kappa carrageenan using vinyltriethoxysilane as a silane crosslinker and KPS as an initiator synthesized a hydrogel network structure [93]. Elvira et al. prepared novel biodegradable hydrogels of acrylamide and acrylic acid in the presence of starch using the free radical mechanism. The hydrogels showed desirable characteristics, such as pH sensitivity, biodegradability, and swelling properties [94].

**Figure 8:**
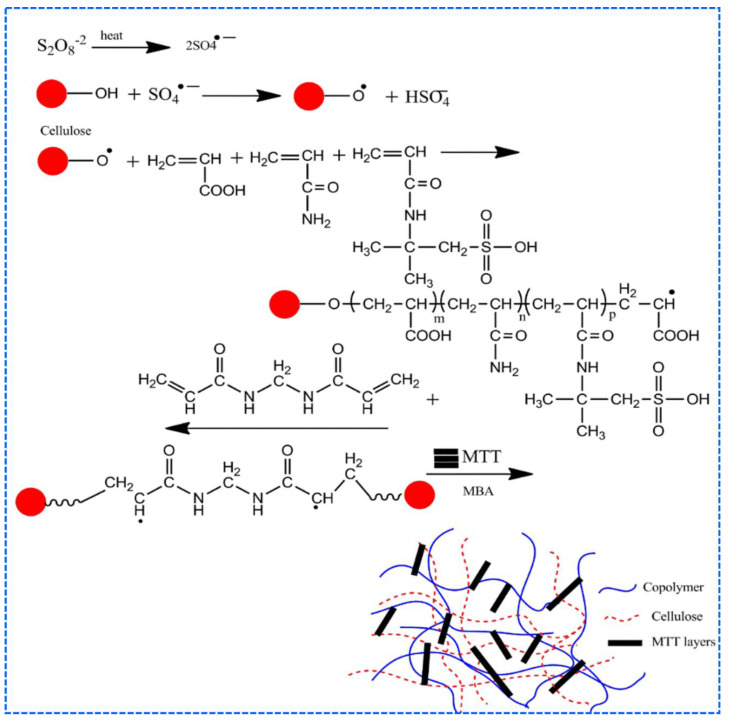
The plausible mechanism of carboxymethyl cellulose-g-poly (acrylamide-co-acrylic acid-co-2-acrylamido-2-methyl-1-propanesulfonic acid (AMPS)/montmorillonite (MMT)) hydrogel synthesis [95].

Bao et al. reported superabsorbent hydrogels of sodium carboxymethyl cellulose-graft-poly (acrylamide-co-acrylic acid-co-2-acrylamido-2-methyl-1-propanesulfonic acid (AMPS))/montmorillonite (MMT)) using KPS and MBA as an initiator and a crosslinking agent, respectively. These super porous hydrogels contained porous networks owing to MMT and carboxymethyl cellulose, with side chains carrying carboxamide, carboxylate, and sulfate. The swelling ratio of the hydrogels exhibited high external pH sensitivity. The hydrogels showed swelling ratios in the following order in the presence of salt solutions: K^+^ > Na^+^ > Ca^2+^ > Mg^2+^. The plausible mechanism of hydrogel synthesis is shown in Figure 8 [95].

### 5.3. Interpenetrating Network (IPN) Hydrogel

**Figure 9:**
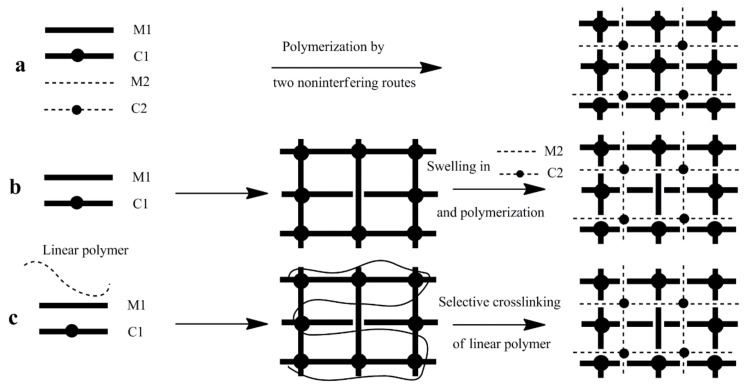
Proposed reaction mechanism of interpenetrating network (IPN) formation: (**a**) simultaneous strategy; (**b**) sequential strategy; (**c**) selective crosslinking of a linear polymer entrapped in semi-IPN [96].

Crosslinked polymer networks can be supplementarily reinforced by interlocking secondary polymers within the entangled networks. A polymer comprising of two or more networks, which are at least partially interlaced at a molecular scale but not covalently bonded to each other and cannot be separated unless chemical bonds are broken, is known as an interpenetrating polymer network (IPN). In other words, when the entangled polymer networks are allowed to swell in an aqueous solution in the presence of polymerizable monomers, and these monomers polymerize to form physically associated networks, they are called interpenetrating polymer networks (IPNs). There are two different possibilities of IPN formation—if one polymer network is crosslinked while the other polymer physically associated with the crosslinked polymer, the network formed is called a semi- interpenetrating polymer network (semi-IPN). However, if the second polymer is also crosslinked with the already crosslinked polymer network, then the network formed is called a full-interpenetrating polymer network (full-IPN). Numerous polymers form these types of networks in order to balance the deficiency of another polymer. These polymers may be natural polysaccharides, proteins, or synthetic hydrophilic polymers containing CONH_2_, OH, SO_3_H, COOH, NH_2_, and quaternary ammonium groups. IPN hydrogels can be prepared via three different routes by combining of natural polymers as well as synthetic polymers. These routes are presented in Figure 9.

According to the chemistry of preparation, IPN hydrogels can be classified in: (i) simultaneous IPN, when the precursors (M1, C1, M2, and C2) of both networks are mixed and the two networks are synthesized at the same time by independent, noninterfering routes such as chain and stepwise polymerization (Figure 9a), and (ii) sequential IPN, typically performed by swelling of a single-network hydrogel into a solution containing the mixture of monomer, initiator and activator, with or without a crosslinker (Figure 9b). If a crosslinker is present, full-IPN results, while in the absence of a crosslinker, a network having linear polymers embedded within the first network is formed (semi-IPN). When a linear polymer, either synthetic or biopolymer, is entrapped in a matrix, thus forming a semi-IPN hydrogel, a full-IPN can be prepared after that by selective crosslinking of the linear polymer chains (Figure 9c) [96].

#### 5.3.1. Semi-Interpenetrating Network (semi-IPN) Hydrogels

**Figure 10:**
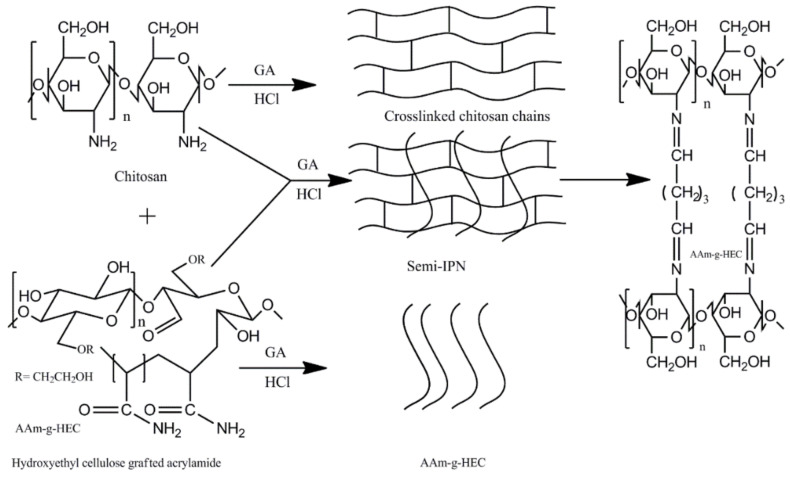
The proposed reaction mechanism of a semi-IPN hydrogel of chitosan/acrylamide-g-hydroxyethyl cellulose [100].

Numerous attempts have been made to synthesize semi-IPN hydrogels of polysaccharides and synthetic polymers for efficient biomedical applications. Semi-IPN chitosan-based hydrogels have been reported by several researchers. In these hydrogels, cellulose and its derivatives and acrylamide-graft-dextran have been blended with chitosan followed by selective chitosan crosslinking through glutaraldehyde [97,98,99,100,101]. Diclofenac sodium was loaded in the semi-IPN hydrogels of crosslinked chitosan entrapped with acrylamide-graft-hydroxyethyl cellulose. The drug loading efficiency was found to range from 50 to 66%. The schematic representation of crosslinked chitosan entrapped with acrylamide-g-hydroxyethyl cellulose is shown in Figure 10.

Yang et al. prepared semi-IPN hydrogels comprised of poly (ethylene glycol) grafted on alginate and carboxymethyl chitosan for the oral delivery of protein in the intestine. They found that the hydrogels were promising in protein delivery [102]. Semi-IPN hydrogels of chitosan and its derivatives with numerous ionic polymers, including cationic groups, such as amine and quaternary ammonium groups, and anionic groups, such as carboxylic groups, were synthesized. These hydrogels were synthesized by selective crosslinking of polyelectrolytes in the presence of chitosan or crosslinking of chitosan in the presence of pre-formed polyelectrolytes. The studies revealed that ionic interaction of COO^−^ ions of polyelectrolytes and NH_3_^+^ from chitosan resulted in an increase in mechanical strength of the hydrogels but a decrease in the swelling ratio of the gels. These interactions increased the crosslinking intensity of the hydrogels. These hydrogels showed reversible responses to solutions of various pH levels and strengths of salt. These hydrogel systems were found to be more efficient for the controlled release of drugs. All these improvements attracted researchers towards such systems [53,103,104,105,106,107,108,109,110,111]. Various semi-IPN hydrogels based on chitosan and non-ionic polymers have also been reported. These semi-IPN hydrogels were synthesized through either crosslinking of chitosan in the presence of un-crosslinked nonionic monomers or nonionic monomers that were crosslinked in the presence of chitosan solution. The nonionic monomers mostly consisted of acrylamide and its derivatives and 2-hydroxyethyl methacrylate. Swelling kinetics studies revealed that all gels followed the Fickian diffusion mechanism for water transport [112,113,114,115,116]. Modulations in mechanical strength and water contents were investigated in all these hydrogels. The mechanical strength and water contents were dependent on the composition of hydrogels. Other factors might be the use as a controlled release carrier and scaffolds in tissue engineering.

Several semi-IPN hydrogels comprised of alginate and synthetic polymers have been investigated. These hydrogels had novel properties, such as outstanding porosity, sustained drug delivery, multi-responsiveness, and electrical sensitivity. Alginate- and methacrylic acid-based semi-IPNs revealed a significant response to electrical field. Therefore, it was suggested that the hydrogels should be used in artificial organ components, sensors, and electrical sensitive drug delivery systems [117]. Amphoteric semi-IPN hydrogels have been prepared through graft copolymerization of acrylic acid on cationic starch in the presence of poly (methacryloyloxyethyl ammonium chloride) (PDMC). The salt link between quaternary ammonium groups of PDMC and COO^−^ ions of starch-graft-poly (acrylic acid) were investigated by FTIR analysis. PDMC was entrapped within the starch-graft-poly (acrylic acid) hydrogel and was not washed away during the washing process. The hydrogels were highly sensitive to pH, but with the increase in concentration of PDMC, the swelling ratios were decreased in the basic medium due to unavailability of carboxylic groups [118]. Other polysaccharides, such as cellulose, kappa carrageenan, hyaluronic acid, guar gum, and xanthan, also form semi-IPN hydrogels. Semi-IPN hydrogels of kappa carrageenan and poly (N, N′-diethylacrylamide) were reported. These hydrogels were highly temperature-sensitive [119].

#### 5.3.2. Full-Interpenetrating Network (Full-IPN) Hydrogels

When a linear polymer, either a natural or synthetic polymer, is entrapped in the other matrix to form a semi-IPN hydrogel, a full-IPN may be formed by selective crosslinking of a second polymer which is not already crosslinked. Formation of full-IPN can prevail over the thermodynamic incompatibility because stable network formation and limited phase separation can be attained. The interlocked components of crosslinked network structures ensure the stability of full-IPN polymers in the bulk and surface morphology. The major advantages of full-IPN are the relatively compact hydrogel structures with improved mechanical strength, tunable physical properties, and enhanced drug loading efficiency as compared to conventional hydrogels. The surface chemistry and pore size of full-IPNs can be controlled to adjust the drug release rate and interaction between hydrogels and tissues of the body. Numerous natural and synthetic polymers can form full-IPNs, such as chitosan, PVA, etc. The particular characteristics of this new material can be investigated by mechanical strength and resistance to wear. Fang et al. synthesized full-IPN hydrogels composed of chitosan/PNIPAM. In these hydrogels, NIPAM was crosslinked with N, N′-methylenebisacrylamide while chitosan crosslinked with formaldehyde. They studied the properties of hydrogels, including extraction of PNIPAM from crosslinked chitosan networks, phase transition behavior, and the swelling ratio measurements in water and in ethanol/water mixtures. The results obtained revealed dissimilarities from the semi-IPN of chitosan/PNIPAM hydrogel. However, this hydrogel was found to be as thermo-sensitive as semi-IPN chitosan/PNIPAM hydrogel and showed transparency at 30 °C but became opaque above this temperature [120]. Poly (vinyl alcohol) is a hydrophilic polymer, having potential applications in the biomedical field, absorption of heavy metal ions, and removal of toxic dyes from the wastewater. Kim et al. prepared and measured free water contents as well as swelling ratio of a full-IPN hydrogel based on chitosan/PVA using UV irradiation. The results revealed fast swelling of the prepared hydrogel that reached equilibrium within one hour. The free water contents and swelling ratio were increasing with increase in the concentration of chitosan. The hydrogels also exhibited pH- and temperature-sensitive swelling behavior, which revealed significant applications in the biomedical field [121]. Usually, nonporous hydrogels swell very slowly in aqueous phase and also show low drug loading efficiencies. These drawbacks of such hydrogels limit their exploitation in the biomedical field. Therefore, super porous hydrogels restraining carboxymethyl chitosan/poly (acrylamide-co-acrylic acid) hydrogels were prepared to improve the mechanical strength, mucoadhesive force, and drug-loading efficiency of the hydrogels. The swelling ratio of the hydrogels decreased with increasing the concentration of carboxymethyl chitosan, glutaraldehyde amount, and crosslinking time. However, by the formation of a full-IPN structure, the mechanical strength, mucoadhesive force, and drug-loading efficiency of hydrogels were considerably enhanced. These full-IPN hydrogels were biocompatible. These characteristics of hydrogels suggested potential exploitation in mucosal drug delivery systems [122].

Dragan et al. prepared semi-IPN hydrogels of chitosan/polyacrylamide, in which chitosan was entrapped in the polyacrylamide matrix. Moreover, full-IPN hydrogels were synthesized by selective crosslinking of chitosan with epichlorohydrin (ECH). This crosslinking was performed in an alkaline medium, and simultaneously, an amide group of polyacrylamide matrix was partially hydrolyzed to generate anionic sites. FTIR analysis demonstrated the partial hydrolysis of the amide group and confirmed the formation of a full-IPN structure [123].

Yin et al. prepared super porous IPNs composed of acrylamide, sodium acrylate, and sodium alginate. Acrylamide and sodium acrylate were crosslinked in the presence of sodium alginate. The CaCl_2_ was added to crosslink sodium alginate in the semi-IPN hydrogels and sodium bicarbonate was used as a blowing agent. These super porous hydrogels showed fast water uptake, high swelling ratio and pH sensitivity, and biocompatibility and had good mechanical properties [124]. Other full-IPN alginate hydrogels have also been reported in the literature [125,126]. Anionic and cationic groups containing IPN hydrogels are not only stabilized by covalent linkages but also by ionic interaction of these groups as well. These ionic interactions improve the mechanical strength and promote the reversible pH and ionic strength responsive in hydrogels. These hydrogels form polyion complexes due to the presence of opposite charges. These types of hydrogels are a very interesting category, having significant potential applications as biomaterials.

## 6. Properties of Hydrogels

The use of polymers (natural or synthetic) containing hydrophilic pendant groups to synthesize hydrogels for biomedical applications is greatly advantageous because these hydrophilic groups not only facilitate ample water absorption but also assist in the interaction with biological tissues (epithelial tissues and mucous membranes). Normally, hydrogels in the fully swollen state are nearly viscoelastic, soft, rubbery, and low in interfacial angle with biological fluids, which decreases the chances of a negative immune response. All these factors contribute to the biocompatibility of hydrogels. The hydrogels are also normally degradable to different extents, depending upon the type of crosslinker involved.

Furthermore, hydrogels have a swelling property, which is the most significant one in their existence. The swelling of hydrogels takes place in three steps: (i) diffusion of water into the hydrogel network (water moving in is called primary bound water), (ii) relaxation of polymer chains (more water moving in is called secondary bound water), followed by (iii) expansion of the hydrogel network (additional water moving in is termed as free water). According to the Flory–Reihner theory, swelling is a function of the elastic nature of the polymer chains and their compatibility with water molecules [127].

Hydrogels show different responses to changes in environmental stimuli, which may generally be categorized into (i) physical (temperature, light, etc.), (ii) chemical (pH and ionic strength), and (iii) biological (enzymes) stimuli. Chemical and biological stimuli are internal, whereas physical stimuli are external, except for temperature, which may be external or internal. Besides all this, there is a special type of stimulus-responsive smart hydrogel called shape memory hydrogel, with two characteristics: (i) permanent shape and (ii) a chemical or physical code that can help restore its original shape [128].

Covalently crosslinked hydrogels that contains ionic pendant groups show a response to change in pH and are referred to as pH-responsive hydrogels. Hydrogels with anionic groups swell at a higher pH due to ionization of pendant groups, which causes electrostatic repulsion. However, hydrogels containing cationic groups swell at a low pH. Some hydrogels show swelling/deswelling behavior in response to temperature changes as a result of changes in polymer–polymer or water–polymer interactions and are known as temperature-sensitive hydrogels. Poly (N-isopropylacrylamide) (PNIPAM) hydrogel is an example of a temperature-sensitive hydrogel [129]. Hydrogels may be positively temperature-sensitive, showing swelling at a higher temperature than the upper critical solution temperature (UCST) [130], negatively temperature-sensitive, showing swelling at a lower temperature than the lower critical solution temperature (LCST), or thermally reversible hydrogels that show sol-gel transition above and below a characteristic temperature. Poloxamer hydrogels are known as thermally reversible hydrogels [131].

### 6.1. Mechanical Properties

The mechanical strength of hydrogels is incredibly imperative for pharmaceutical and biomedical applications. The assessment of mechanical strength of the hydrogels is necessary and significant for suitable physiological function in various perspectives, such as for biomedical applications, tendon and ligament repair, replacement of cartilage, tissue engineering, wound dressing, and drug delivery matrix. It is significant for the hydrogel to sustain its physical texture during release of therapeutic agents for a specific time period.

The preferred mechanical properties of hydrogels may be attained by incorporating specific polymers, co-monomers, and crosslinkers and by changing the crosslinking degree. A strong gel network can be obtained with increasing the degree of crosslinking [132]. However, too high of a degree of crosslinking will result in low elongation and elasticity with greater brittleness. Elasticity is very significant to give increased flexibility to the crosslinked network and to assist in the movement of incorporated therapeutic moieties. Therefore, an optimal degree of crosslinking for hydrogels is essential in order to retain the compromise between mechanical strength and elasticity [133].

Evaluation of the mechanical properties of hydrogels can be done through numerous techniques, such as compression and tension analysis, which can be done by confined or unconfined local indentation with a probe or frequency-based tests using rheometry and dynamic mechanical analysis. Frequency-based sinusoidal testing is usually done using a rheometer. The sample is loaded on a specified geometry instrument and different types of sweep measurements can be performed [132,134].

The viscoelastic properties of hydrogels can be investigated by a rheometer. Rheology may be defined as “the science of deformability and flow of matter under stress or strain. It comes from the Greek words “rheo”, meaning flow, and “–ology”, for study of [135]. This definition was acknowledged by the American Rheology Society in 1929. Over the past three centuries, ideal liquid and solid behaviors were characterized mathematically by Newton’s and Hooke’s laws, respectively. However, many materials possess a transitional behavior between perfect liquids and solids. These behaviors cannot be explained by these classical theories. Scientists felt doubts after the non-ideal behaviors of fluids and silk thread described by Maxwell and Wilhelm Weber, respectively. According to Maxwell, fluids showed elastic properties, which were verified mathematically. Moreover, Wilhelm Weber found non-ideal elastic behavior of silk thread. These materials were typically classified as viscoelastic and non-Newtonian materials. Viscoelastic materials can be recovered after a small deformation, while the viscosity of non-Newtonian materials is stress- and time-dependent [136].

The variable of shear stress and strain are important parameters in rheology. Shear stress is defined as “the ratio of force per unit area to cause disorder in the materials between plates”. Strain is “the ratio of deviation in displacement (x) of material to the height of the material (h) or more simply is tan α”. After applying force, the velocity of the movement is controlled by the internal force acting within the material. The mechanical properties of hydrogels can be found by small deformation rheology experiments. When a hydrogel is deformed by applying force, the material particles are displaced relative to each other, causing strain. Moreover, it is well-known that elastic materials undergo changes in shape on applying force, resulting in elastic strain, and return to their original shape when the force is removed. In contrast, an external force applied on viscous materials causes irreversible strain. This input force causes the viscous materials to flow. However, hydrogels are neither fully viscous nor completely elastic but show a viscoelastic nature. Rheological experiments are conducted within the linear viscoelastic region (LVR) of the investigated materials. The LVR ensuring the mechanical properties of hydrogels is not affected by the magnitude of the imposed stress or strain. Moreover, deformations in viscoelastic materials by applying slow external force are still very small. This is owing to the molecular arrangements in the polymers, which are still close to equilibrium. The mechanical behavior is, then, just a reflection of dynamic processes at the molecular level, which go on constantly, even for a system at equilibrium.

**For controlled-strain rheometers**, shear strain is defined as a sinusoidal function of time (t) which can be expressed as in Equation (1):

γ_(t)_ = γ_0_ (sin ωt) (1)

where γ_0_ is the magnitude of applied strain and ω is the angular frequency of oscillation. It is measured in rad/s. The relation between angular frequency and frequency measured, f, in Hertz is ω = 2 πf. Furthermore, the sinusoidal strain causes sinusoidal stress, which can be illustrated as in Equation (2).

τ_(t)_ = τ_0_ (sin ωt + δ) (2)

where τ_0_ is the stress amplitude and δ is the difference in phase between two waves.

**For controlled-stress rheometers**, shear stress is defined as a sinusoidal function of time (t), which can be expressed as in Equation (3):

τ_(t)_ = τ_0_ (sin ωt) (3)

Sinusoidal stress causes sinusoidal strain, which can be illustrated as in Equation (4).

γ_(t)_ = γ_0_ (sin ωt + δ) (4)

Purely elastic materials follow Hooke’s law, which states that stress and strain are always in phase. So, the phase angle is 0 (δ = 0°). On the other hand, pure viscous materials have two different waves, having phase angle 90° (δ = 90°). However, viscoelastic materials have a phase angle somewhere in between 0 and 90°.

In small-amplitude oscillatory measurements, the shear storage modulus (G’), loss modulus (G”), and loss factor, tan δ, are significant properties of hydrogels. The storage modulus evaluates the deformation energy stored during the process of shearing of a material which expresses its rigidity. The loss modulus measures the energy dissipated during shearing which characterizes the flow behavior of material. The loss factor, tan δ, is the ratio of G” and G’ (G”/G’). When tan δ is greater than 1 (G” > G’), materials have more viscous characteristics. On the other hand, tan δ < 1 (G’ > G”) means materials possess more elastic solid characteristics. The storage modulus and loss modulus are examined as a function of strain, time, and frequency. The gelation process can be investigated by observing the temporal assessment of storage and loss modulus. The LVR within which both moduli are independent of strain can be determined by observing these moduli of the material as a function of strain.

The behavior of a hydrogel at different time scales can be investigated by measuring the moduli against frequency. When G’ >> G” at high frequency for short time, the material has quite solid characteristics, and when G” > G’ at low frequency for long time, then the material has dominant liquid behavior. Moreover, gelation kinetics and gel rigidity are very important factors which have significant influences on the material’s application.

**Figure 11:**
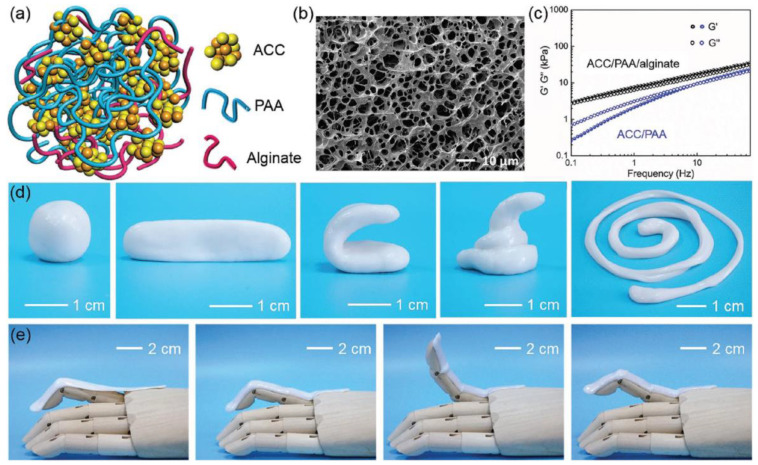
(**a**) Schematic structure of the ACC/PAA/alginate mineral hydrogel. (**b**) SEM image of the freeze-dried ACC/PAA/alginate hydrogel. (**c**) Frequency dependencies of the storage (G’) and loss (G″) moduli of the ACC/PAA/alginate and ACC/PAA hydrogels. (**d**) The ACC/PAA/alginate hydrogel can be manipulated into various shapes. (**e**) When a hydrogel film is attached to a prosthetic finger, it dynamically adapts to the highly nonlinear surface and accommodates the finger movements [139].

The mechanical properties of viscoelastic materials are independent of strain up to a certain value known as critical strain. When strain increases above this value, the storage modulus declines and a non-linear behavior is observed. The strain-dependent G’ and G” measurements are the first step to investigate the LVR of a material at a frequency of 1 Hz. Further, the structure of a material can be investigated at a constant strain below the critical level. These observations provide more information about the effect of colloidal forces and interactions among particles. Below the critical strain, G’ is independent of frequency, which shows the characteristics of solid materials. Conversely, frequency-dependent G’ represents the fluid-like material. Jeong et al. observed the sol-gel behavior of thermogelling chitosan-graft-(PEG-PAF). The storage and loss moduli were examined against frequency at 10 and 37 °C. It was observed that G” was higher than G’ (sol behavior) at 10 °C while G’ was higher than G” (gel behavior) at 37 °C. However, the storage modulus was independent of frequency [137]. The gelation mechanism and the stability of the gel material during the testing time and for significant exploitation at commercial level are other aspects. For this purpose, time sweep profiling of the materials at critical strain and at a frequency of 1 Hz can be carried out. Moura et al. investigated the gel kinetics and gel properties upon crosslinking of chitosan through genipin. The moduli results revealed the dominance of viscous behavior when the chitosan was in solution state. Upon addition of a crosslinker (genipin), the elastic modulus increased due to the crosslinked gel network. It was also observed that the gelation time decreased owing to the increase in crosslinker concentration [138]. Recently, Lei et al. reported a novel mechanically compliant and autonomous mineral hydrogel that was composed from poly (acrylic acid) (PAA) and sodium alginate and physically crosslinked by amorphous calcium carbonate (ACC). The schematic diagram and well-connected porous surface morphology are shown in Figure 11a,b. In addition, sodium alginate was introduced to form strong physical interactions with the Ca^2+^ ions from ACC. This could be investigated from the rheology results of ACC-incorporated poly (acrylic acid) (PAA), where the loss modulus (G″) was higher than the storage modulus (G’) while ACC physically crosslinked PAA/sodium alginate hydrogels, the storage modulus was higher than the loss modulus, revealing the dominant behavior of elasticity over viscous nature (Figure 11c). The mechanical flexibility of the hydrogels can also be observed from the different shapes of the hydrogels in Figure 11d. When this hydrogel film was attached to a prosthetic finger, it dynamically adapted to the highly nonlinear surface and accommodated the finger movements owing to the flexible characteristics [139].

**Figure 12:**
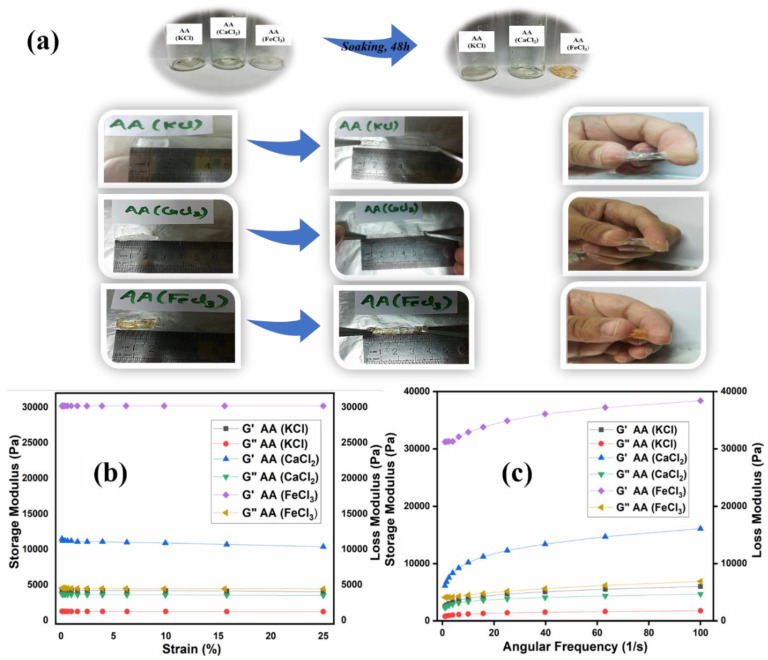
(**a**) Mechanical strength of the hydrogels, (**b**) strain amplitude test, and (**c**) frequency sweep study [140].

Khan et al. observed the mechanical strength in terms of strain amplitude and frequency sweep of poly (acrylic acid) hydrogel electrolytes. The results revealed the viscoelastic response of the hydrogels and storage modulus was higher than loss modulus and hydrogels were highly elastic. This elasticity was owing to the carboxylic acid groups present and the interaction between cations from the salt and carboxylate anion. In addition, mechanical strength of the hydrogel was dependent on the extent of coordination between carboxylate anion and metal cation. The mechanical strength of the hydrogel is shown in Figure 12 [140].

### 6.2. Biocompatibility

The cytotoxicity of a biomaterial is very important in order to make it applicable in the biomedical field. Similarly, hydrogels should be biocompatible and non-toxic. Basically, biocompatibility is the property of material to work efficiently with the host tissues and respond properly in an explicit environment. Bio-safety and bio-functionality are two basic elements of biocompatibility. If the hydrogels cannot fulfill these conditions, then they can be fouled. Toxic chemicals used in the preparation of hydrogel formulations often cause challenges for in vivo biocompatibility. However, polysaccharides are widely regarded as being non-toxic, biologically compatible polymers and are approved for dietary applications. Tokura et al. reported in vivo cytotoxicity studies of different biodegradable polysaccharides. No acute toxicity was noticed in rat blood systems [141]. Chan et al. investigated the cytotoxicity of chitosan, chitosan-PEG, and folic acid-conjugated chitosan-PEG/DNA complexes. The results revealed an average cell viability of 90% [142]. Another study showed the in vitro cytotoxicity profiles of chitosan, O-carboxymethyl chitosan, and N, O-carboxymethyl chitosan to breast cancer cells MCF-7. The results showed that these are much less toxic and the cells had 98% viability [143]. NSC is a well-known biocompatible material and, like chitosan, can be used in the preparation of hydrogels, microparticles, nanoparticles, and nanospheres. Lu et al. observed the cytotoxicity of NSC/carboxymethyl cellulose hydrogel on the proliferation of HEK 293T cells. The results demonstrated 93% cell viability after 48 h [144]. Moreover, cytotoxicity of NSC hydrogel formulations in 293T, CHO, HepG2, and HeLa cells have been investigated and researchers found the non-toxic nature of NSC [145,146]. Chitosan is non-toxic and tissue-compatible, but NSC is a synthesized derivative and is known to significantly promote cell proliferation. Zhu et al. assessed in vitro cell toxicity and found that NSC had no negative effects on 3T3 fibroblasts up to 0.25 mg/mL [147]. Furthermore, Mishra et al. did the MTT assay of pectin/poly (vinyl pyrrolidone) (PVP) hydrogel membranes on B16 melanoma cells. The results showed that the hydrogel membranes did not induce significant cytotoxic effects even at high concentrations of polymer solution. This indicates the biocompatibility of the hydrogel membranes [148]. Some synthetic polymers are non-toxic and biocompatible with living cells. The cytotoxicity of poly glutamic acid, poly (acrylic acid), poly (methacrylic acid), poly (ethacrylic acid), and poly (propacrylic acid) hydrogels was studied on HeLa cells and these hydrogels showed 90% cell viability. Wang et al. investigated the cytotoxicity of methoxyl poly (ethylene glycol)-poly (caprolactone)-acryolyl chloride (PEC-AC) on HEK 293 cells and found that cell viability was decreased with the increase in concentration of PCE-AC. The minimum viability was found to be 60% when the concentration of PEC-AC copolymer was 200 µg/mL [149]. Therefore, most polysaccharides are biocompatible and can be used as biomaterials. Additionally, synthetic polymers are also non-toxic to some extent but show less cytocompatibility compared to natural polysaccharides.

**Figure 13:**
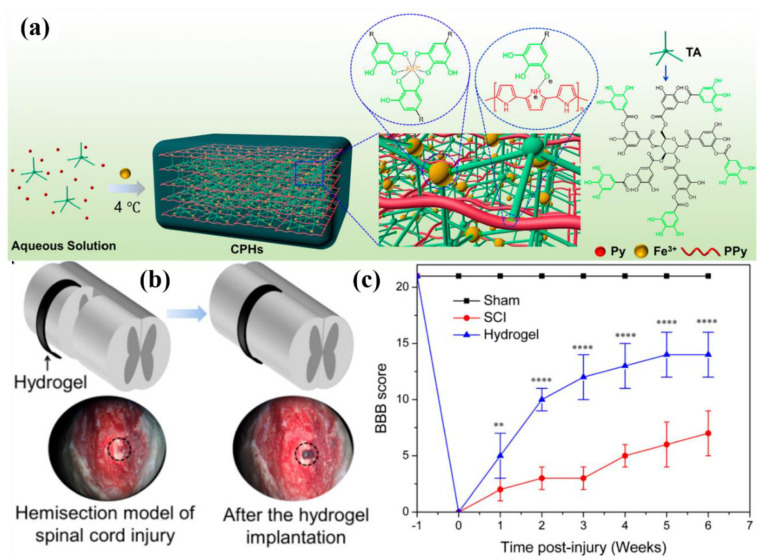
(**a**) Illustration of the fabrication of a crosslinked conducting polymer hydrogel (CPH) following the addition of tannic acid (TA), polypyrrole (Py), and Fe^3+^ ion. TA acts as a crosslinking agent and dopant and Fe^3+^ ion acts as an oxidant and ionic crosslinking agent. (**b**) Graphical representation of a “C”-shape, semi-tubular CPH that was implanted as a bridge to cover the spinal cord hemi-section gap. (**c**) Locomotor recovery of the animals was measured using the standard BBB scale in an open field; ** *p* < 0.01, **** *p* < 0.0001; n = 5 animals in each group. Error bars represent the standard deviation [150].

The human body is directly in contact while working with implantable and electronic devices. Therefore, materials used in these devices should be biocompatible and should not harm human health. Hydrogels are ideal candidates for such devices and can play the role of a bridge between electronics and biology. To investigate the biocompatibility of conducting hydrogels, rabbits were exposed to polyaniline hydrogel scaffolds. The fact manifests that cell morphology was plumper and the diameter of the cell was larger compared to the counterpart without polyaniline hydrogel. Similarly, polypyrrole-conducting hydrogels are also biocompatible and Li et al. synthesized polypyrrole hydrogels using tannic acid as a crosslinking agent as well as a dopant and used it for spinal cord injury. Conducting hydrogels could accelerate neural stem cell differentiation into neurons and activated the locomotor function of endogenous neural stem cells (Figure 13a–c) [150].

**Figure 14:**
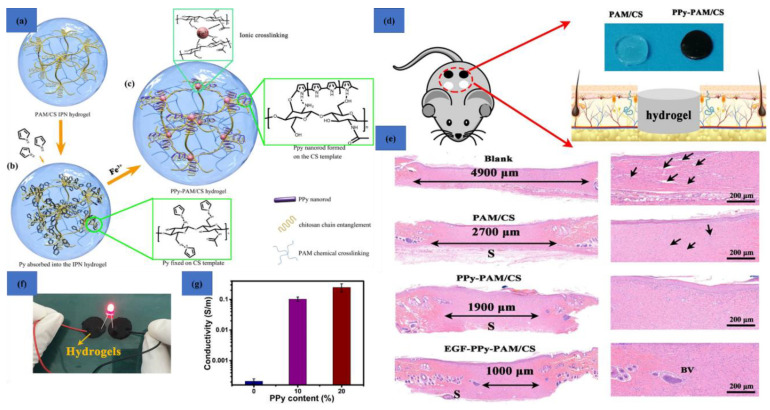
(**a**) Synthesis of polyacrylamide/chitosan (PAM/CS) interpenetrating (PAM/CS IPN) hydrogels; (**b**) polypyrrole was absorbed into the IPN hydrogel, fixed on CS chains, and accumulated in the zone of CS entanglement; (**c**) polypyrrole (PPy) was polymerized in situ in the hydrogel under the controlling of CS molecular templates; PPy-conductive pathway (purple line) intertwisted along CS chains (purple line), and PPy nanorods aggregated on the chain entanglement zone of CS hydrogels for repairing full thickness defects on rats. (**d**) Schematics of hydrogel implantation and conductive properties of the hydrogel. (**e**) H&E (histomorphological evaluation) staining of wound sections after 21 days; S, sample; BV, blood vessel. (**f**) PPy–PAM/CS hydrogel was connected to a circuit and illuminated an LED; (**g**) conductivity of the PPy–PAM/CS hydrogel with different contents of PPy [151].

Gan et al. developed conducting hydrogels of polypyrrole, chitosan, and polyacrylamide. This conducting hydrogel could exhibit excellent results in drug delivery, wound healing, and cell adhesion. The conducting hydrogel exhibited conductivity as well as electrochemical application in terms of lighting up of a light-emitting diode (LED) (Figure 14a–g) [151].

### 6.3. Biodegradability

The exploitation of biodegradable hydrogels is essential in the biomedical field. Biodegradability means the breakdown of hydrogels into harmless end products by the living organism. The biodegradability of hydrogels depends on the moieties present in the systems and the method of preparation. The processes of degradation include hydrolysis and solubilization of biological entities of hydrogels into end products. The hydrogels may degrade and be eliminated from the body through bio-absorption and bio-erosion.

Numerous hydrophilic natural and synthetic polymers can be classified as biodegradable polymers. These polymers absorb water due to diffusion and swell up to large extent and eventually dissolve into water upon auxiliary water absorption. The degradation of these polymers depends on several factors, such as hydrophilicity, molecular weight, and polymer–water interaction. Other environmental factors, such as pH and temperature, may also control the degradation through simple solubilization. However, this class of polymer is very limited. There are a range of polymers which cannot be degraded by simple hydrolysis but can be degraded through chemical hydrolysis. These polymers do not form hydrogels but combine with hydrogel, forming hydrophilic monomers or polymers to form biodegradable hydrogels. These hydrogel systems usually undergo degradation through chemical hydrolysis of ester linkages. Moreover, hydrogels can also be degraded through enzyme hydrolysis, and this class of hydrogels includes polymers, for instance, polysaccharides, proteins, and synthetic polypeptides. Due to the significant advantage of enzymatic degradation, these polymers can be used in drug delivery systems. Enzyme hydrolysis occurs through the group of hydrolases which catalyze the hydrolysis of C-C, C-O, and C-N bonds. Hydrolases that degrade protein and polypeptide hydrogels are proteinases and peptidases, respectively. Furthermore, the biodegradation of polysaccharide hydrogels only occurs through glycosidase. Specifically, chitosan can be degraded by enzyme hydrolysis of glucosamine-glucosamine, glucosamine-N-acetyl-glucosamine, and N-acetyl-glucosamine-N-acetyl-glucosamine. However, the degradation of chitosan also occurs through free radical and redox reactions. Lysozyme had been used to study the in vitro degradation of chitosan. It was found that 50% acetylated chitosan showed a 66% viscosity loss in first 4 h of incubation in a buffer solution of pH 5.5 [152]. Modification of chitosan causes no significant change in the rate of degradation. Trimethylchitosan showed degradation at a similar rate to chitosan but the rate of degradation was not sensitive to pH [153]. In vitro degradation of tripolyphosphate- and glutaraldehyde-crosslinked chitosan films were investigated by McConnell et al., where porcine pancreatic enzymes were used to investigate the degradation rate. The results revealed that glutaraldehyde-crosslinked films showed higher rates of degradation than tripolyphosphate-crosslinked hydrogels. Furthermore, NSC has considerable biodegradability in vitro as well as in vivo. Normally, the degradability of a hydrogel depends upon the pH, temperature, oxygen content, water potential, structure of the polymer network, crystalline nature of the polymer, and the type of linkages. Degradability is observed by a change in weight over a specific period of time. The biodegradability of NSC was confirmed using an NSC/OCMC hydrogel in a phosphate buffer solution at 37 °C. In this hydrogel, Schiff-base linkages were hydrolytically susceptible and not stable, resulting in an enlarged lattice size of the network. This phenomenon led to disintegration of the hydrogel [154]. Tan et al. also studied biodegradation of an NSC/PEG hybrid hydrogel in phosphate buffer solution at 37 °C. The results revealed that the hydrogel was biodegradable. Other polysaccharides, such as starch [155], cellulose, dextran [156], alginate, pectin [157], and natural gum hydrogels [158], are also as biodegradable as chitosan and its derivatives. Some synthetic polymers are also biodegradable but the rate of degradation is very low as compared to natural polymers [159]. Gao et al. studied the in vitro biodegradation of poly (acrylic acid) and its derivatives and poly (glutamic acid) in buffer solutions of pH 1.2 and 7.4 in the presence of proteinase. The results revealed a higher degradation rate at pH 7.4 and lower degradation at pH 1.2. This might be due to higher hydrophilicity at pH 7.4 and higher hydrophobicity at pH 1.2 [160].

### 6.4. Swelling Behavior of Hydrogel

**Figure 15:**
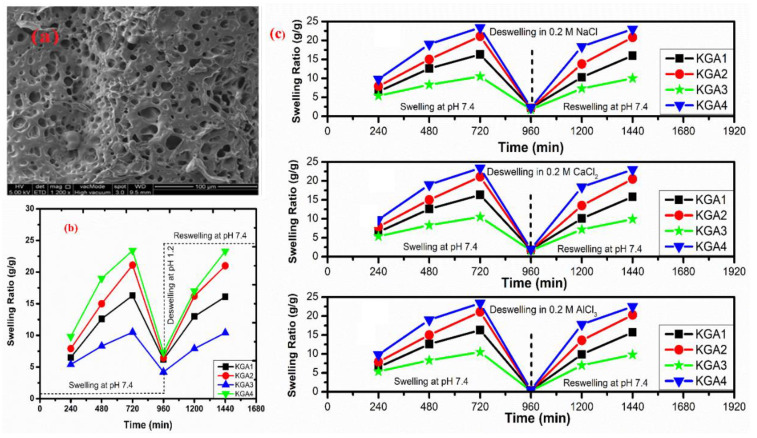
(**a**) Morphology of karaya gum-g-poly (acrylic acid) hydrogel, (**b**) swelling–deswelling–reswelling at different pH levels, and (**c**) swelling–deswelling–reswelling in salt solutions [161].

The swelling response in different matrix environments, such as water, pH, and ionic strength, is the characteristic of hydrogel to be used in different fields. The hydrogel responds to its biological and environmental media, such as pH, ionic media, solvent, electric field, exposure to light, and temperature [161]. The swelling kinetics and equilibrium are affected by different factors, such as crosslinking ratio, chemical nature of polymers, ionic media, and synthesis state. Swelling is measured in terms of swelling ratio, which is the weight swelling ratio of swollen gel to dry gel. Crosslinking affects the swelling ratio of hydrogel, as highly crosslinked structures have a lower swelling ratio and vice versa. Chemical structure also has a significant function to the swelling property due to the hydrophilic and hydrophobic groups present on the polymer chains. Hydrogels containing more hydrophilic groups swell more as compared to hydrophobic groups. The swelling of hydrogels is also affected by temperature and pH. pH-sensitive hydrogels swell due to the ionization of hydrophilic groups with variation in pH levels. Ionization produces electrostatic repulsion between like charges on the polymer and breaks the secondary bonding between polymer chains. Swelling of hydrogels involves three steps: first is the diffusion of water into the hydrogel network; second is the loosening up of polymer chains; third is the expansion of hydrogel network. Hydrogel in a dehydrated state is referred to as the glassy state, and in swollen form, as the rubbery state. Free spaces in the chains permit the solvent molecules to find spaces when glassy or dry hydrogel makes contact with the aqueous medium. When enough water has entered into the hydrogel matrix, the glassy state turns into the rubbery state, named as swelling. The diffusion process is responsible for the entrance and removal of water from the hydrogel matrix. Figure 15 shows the morphology and swelling/reswelling properties at different pH levels and in salt solutions. Similarly, Bashir et al. prepared karaya gum-g-poly (acrylic acid) and observed the swelling–shrinkage–reswelling properties of hydrogels at different pH levels and in salt solutions. In addition, water retention was also observed, and results are shown in Figure 15 [161].

**Figure 16:**
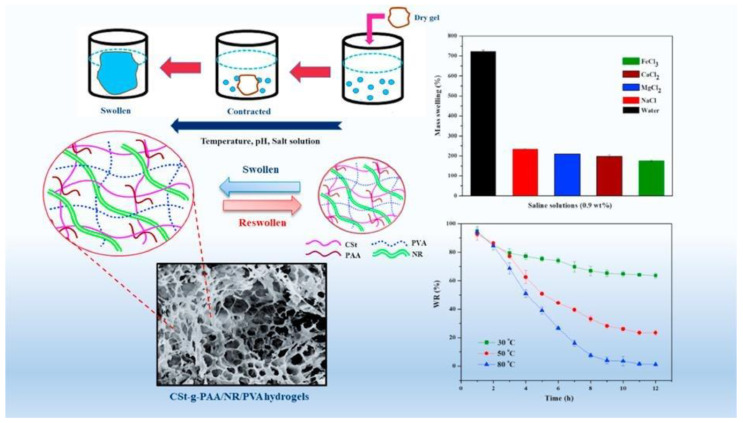
Swelling–reswelling at different pH levels, in salt solutions, morphology, and water uptake of hydrogels [162].

Tanan et al. synthesized semi-IPN of cassava starch-g-polyacrylic acid/natural rubber/polyvinyl alcohol blend (CSt-g-PAA/NR/PVA) hydrogels and observed the swelling/reswelling properties at different pH levels and in salt solutions and water retention (%). The swelling properties were highly sensitive to the salt and pH (Figure 16) [162].

#### Swelling Kinetics

Practically, the swelling of hydrogels follows first-order and second-order kinetics, depending on the rate of swelling with respect to time, the swelling capacity of the hydrogels, the size distribution of the particles, and the polymer gel composition. The diffusion-controlled system follows first-order kinetics. According to this, the rate of swelling at any time is directly proportional to the swelling medium content before reaching the equilibrium of absorbed water (W_e_). The swelling of hydrogels is given in Equation (5).

(5) $$\frac{\mathrm{dW}}{\mathrm{dt}}={\;k}_{f}\;\left(W_{e}-{\;W}_{t}\right)$$

where k_f_ is the rate constant for first-order kinetics, $W_{e}$ is the amount of absorbate at equilibrium, and $W_{t}$ is the absorbate at time t. The above-mentioned equation expresses the swelling of hydrogels in the solvent and shrinkage on drying. The integration of this equation gives the following illustration in Equation (6).

(6) $$\ln\frac{W_{e}}{W_{e}-\;W}=k_{f}t$$

The swelling process follows the first order if the graph between $\ln\frac{W_{e}}{W_{e}-\;W}$ and time t is a straight line. Moreover, polymer systems having limited swelling obey first-order kinetics under these two conditions: the swelling must be diffusion-controlled and obey Fick’s law. However, divergence from first-order kinetics is also possible, specifically at longer swelling times and for extensive swelling of the hydrogel. It is very difficult to follow first-order swelling kinetics throughout the swelling process, especially if the process of polymer relaxation controlled the swelling rather than diffusion. The second-order swelling kinetics equation in terms of swelling rate is described in Equation (7).

(7) $$\frac{\mathrm{dW}}{\mathrm{dt}}={\;k}_{s}\;{\left(W_{e}-{\;W}_{t}\right)}^{2}$$

Rearranging and integrating Equation (7) between the limits when t = 0 to t, $W_{t}=0{\;\mathrm{to}\;W}_{t}$ can be expressed as Equation (8).

(8) $$\overset{W_{t}}{\underset{0}{\int }}\frac{\mathrm{dW}}{{\left(W_{e}-{\;W}_{t}\right)}^{2}}={\;k}_{s}\overset{t}{\underset{0}{\int }}\mathrm{dt}$$

Equation (8) becomes:

(9) $$\frac{t}{W_{t}}\;\;=\;\;\;\frac{1}{W_{e}}\;t+\;\;\frac{1}{W_{e}^{2}k_{s}}$$

Equation (9) may be written as:

(10) $$W=\;\frac{k_{s}\;W_{e}^{2}t\;}{1+\;W_{e}k_{s}t}=\;\frac{r_{o}t}{1+\;W_{e}k_{s}t}$$

where $r_{o}$ is the initial rate of swelling, $r_{o}\;=\;k_{s}\;W_{e}^{2}$.

According to Equation (9), the profile of $\frac{t}{W_{t}}$ versus t should give a straight line with a slope of $\frac{1}{W_{e}}$ and an ordinate of $\frac{1}{W_{e}^{2}k_{s}}$.

### 6.5. Stimuli Sensitive Hydrogels

**Figure 17:**
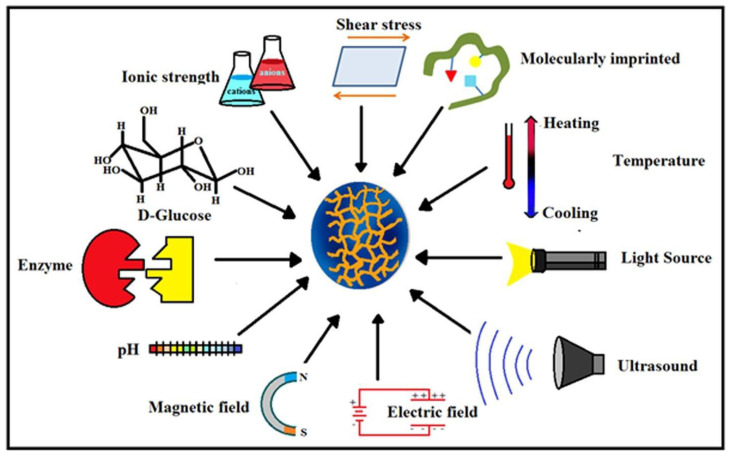
Environmental stimuli sensitive to hydrogels [163].

Recently, stimuli polymer hydrogels have attracted striking attention in numerous fields. These hydrogels respond to environmental changes, such as pH, light, temperature, ionic strength, and electric and magnetic fields. Some of these stimuli-sensitive properties are discussed in this section, and various factors can be seen in Figure 17.

#### 6.5.1. pH-Sensitive Hydrogels

pH-sensitive polymer hydrogels restrain pendant acidic and basic groups. Polymers containing acidic groups, such as carboxylic acid and sulfonic acid, are known as anionic polymers, while polymers consisting of basic groups, such as amines, are known as cationic polymers. These groups accept or release protons and become ionized with changes in pH. Polymers including large numbers of such groups are recognized as polyelectrolytes. Further, three-dimensional crosslinked polyelectrolytes show changes in swelling behavior with changes in pH. The ionization of pendants in polyelectrolytes has a tendency to cause differences in apparent dissociation constants (K_a_) from the corresponding acid or base. The swelling property of crosslinked polyelectrolytes is mainly caused by electrostatic repulsion of the ionized groups. This property is exceedingly influenced by variations in pH, ionic strength, and electrostatic repulsion. pH-sensitive hydrogels have been exploited for targeted and controlled oral drug-release formulations. This is due to the difference in pH in body parts such as the stomach (pH < 3) and the intestine (pH > 7). The difference in pH is sufficient to exploit the use of hydrogels via oral administration. The swelling behavior is maximal and drug release occurs at low pH levels for hydrogels comprised of polycationic polymers due to ionization of pendant groups. Cationic hydrogels are usually used as stomach-specific drug-delivery carriers. The swelling ratio of the N, N′-dimethylaminoethylmethacrylate (DMAEM) and methylmethacrylate copolymer hydrogel was found to be low at a neutral pH but high at acidic pH. Caffeine was loaded in the hydrogel and it was not released at neutral pH but released significantly at pH 3 [164]. Moreover, crosslinked chitosan and polyethylene oxide (PEO) semi-IPN hydrogels demonstrated high swelling at pH 1.2. These hydrogels would be best for stomach-specific delivery of metronidazole and amoxicillin for *Helicobacter pylori* treatment, as these drugs were released in large amounts at pH 1.2 [165]. However, the pendant groups of anionic hydrogels ionize at neutral pH and the hydrogels show a maximum swelling at this pH. Moreover, this type of hydrogel can be used in intestine-specific drug delivery. Anionic polymers are poly acrylic acid, methacrylic acid, pectin, anionic chitosan derivatives, karaya gum, etc. pH-sensitive semi-IPN hydrogels of poly (acrylic acid) and PEO displayed pH-dependent swelling, which was high at neutral pH and low at acidic pH (stomach pH 1.2). The release behavior of some drugs having different water solubility (including nicotinamide, prednisolone, salicylamide, and clonidine HCl) was also tested. The release patterns were significantly correlated with the swelling behavior of the hydrogels. A greater amount of drugs was released at neutral pH and a small amount of drugs was released at pH 1.2 [166]. Moreover, carboxymethyl chitosan crosslinked with glutaraldehyde hydrogels were pH-dependent and promising carriers for colon-specific drug administration [167]. Mura et al. studied colon-targeted delivery of 5-aminosalicylic acid (5-ASA) using chitosan and NSC. They investigated drug release from different matrices of chitosan and NSC. The release rate of 5-ASA from chitosan matrices was fast in acidic medium while the release rate from NSC was rapid in alkaline medium in vitro [168].

#### 6.5.2. Thermosensitive Hydrogels

Temperature is an extensively exploited stimulus in environmentally sensitive hydrogels. Temperature-sensitive polymer hydrogels experience volume phase transformation upon changes in temperature. The effect of temperature on polymer hydrogels can be recognized through direct heating. Polymers increase or decrease their solubility in water with temperature changes. Polymers whose water solubility decreases with increase in temperature show lower critical solution temperatures (LCSTs). However, polymers whose water solubility increases with increase in temperature possess upper critical solution temperatures (UCST). LCST polymer hydrogels swell at low temperatures and shrink at high temperatures exceeding the LCST. These hydrogels are known as negative temperature-responsive systems. Negative temperature-sensitive hydrogels have been used in pulsatile drug-release systems. Poly (N-isopropylacrylamide-co-butyl methacrylate) (poly (NIPAM-co-BMA) hydrogels were synthesized to study pulsatile drug release. The BMA contents caused an increase in mechanical strength. Pulsatile indomethacin delivery was obtained with a continued low temperature but stopped at a high temperature. This release behavior followed the swelling trend of the hydrogels. In another study, poly (N-isopropylacrylamide-co-acrylic acid) hydrogels were synthesized, and the thermo-sensitive property of these hydrogels was shown to be manipulated by changing the molar ratio of the two monomers. Here, 5-FU was loaded with an entrapment efficiency of 4%. The release results clearly investigated the pH- and temperature-dependency of release rates [169]. However, UCST polymer hydrogels display higher swelling with increases in temperature and shrink at low temperatures and are classified as positive temperature-sensitive systems. IPN hydrogels of poly (acrylic acid) and poly (AAm-co-BMA) and crosslinked poly (AAm-co-BMA) and poly (AAm-co-AA-co-BMA) showed positive temperature-responsive swelling behaviors. The study revealed that the increase in hydrophobic butyl methacrylate concentration shifted the transition temperature to a higher temperature. The swelling and drug (ketoprofen)-release rates demonstrated reversible temperature-dependence trends [170]. Another type of temperature-sensitive hydrogel is known as thermoreversible gel. The most common thermoreversible gels are Poloxamer (Pluronics) and Tetronics. The Food and Drug Administration (FDA) and the Environmental Protection Agency (EPA) approved some of these gels to be used in food, pharmaceutical, and agricultural applications. Pluronic F127 20 wt.% solution behaves as a free flowing liquid at temperatures less than 25 °C but turns into a semi-solid gel at a temperature of 27 °C. Cytotoxicity studies revealed the non-toxic nature of Pluronic. Early studies evaluated Pluronic F127 thermo-sensitive solutions for the treatment of burns, topical administration of anti-cancer agents, and sustained delivery of drugs after extravascular parenteral injection [171]. After parenteral injection, poloxamer gels can prolong drug release compared to solutions but the delivery period rarely exceeds a few days. This characteristic makes poloxamer gels interesting for short-term therapies, such as pain management, infection treatment, and fertility control. Moreover, a poloxamer-graft-PAA hydrogel has been reported by Hoffman et al. [172].

## 7. Electrochemical Applications of Polymer Hydrogels

### 7.1. Electrolytes

Besides electrodes, electrolytes also play a major role to transfer and balance charges between the two electrodes. Electrolytes provide high power and energy density, safety, and long cycle life-time of supercapacitors. To improve electrochemical performance, better selection of electrolytes is of key importance. The electrolyte must have the properties of a wide voltage window, high electrochemical stability, small resistivity, and a suitable solvated ionic radius. The electrolyte used in electrochemical supercapacitors can be grouped into four types, which are aqueous electrolyte, organic electrolyte, ionic liquids, and solid and quasi-solid-state electrolytes [173,174,175].

Aqueous electrolytes have a higher conductivity than organic and ionic electrolytes. They have low resistance that can enhance the performance of supercapacitors. Aqueous electrolytes are classified into three groups, which are alkaline, acidic, and neutral. The common solutions used as aqueous electrolytes are KOH, NaOH, LiOH, Na_2_SO_4_, H_2_SO_4_, and NaCl. Many cations from electrolytes, such as Na^+^, H^+^, Li^+^, and K^+^, can affect electrochemical performance. As a result, it shows a difference in the specific capacitance. The difference is due to the mobility and radius of cation and conductivity. If the ionic mobility and conductivity is increased, then the charge transfer becomes faster. In addition, if the moderation sphere radius is too small then the adsorption of ion is higher at the electrode–electrolyte interface. Thus, the smallest moderated ions lead to the highest specific capacitance of supercapacitors. A lot of aqueous electrolytes’, such as 1M Na_2_SO_4_, 1M NaOH, 1M LiOH, 1M KOH, and 6M KOH, solutions were used to study the electrochemical performance of Bi_2_WO_6_ nanoparticles. As a result, KOH electrolyte exhibited the electrochemical performance of Bi_2_WO_6_ owing to their lower equivalent series resistance, small hydration sphere radius and high ionic mobility. It exhibited better coulombic efficiency and specific capacitance. However, aqueous electrolytes have some disadvantages, including low energy density due to the small voltage wind, leakage issue, and cycling stability. Aqueous electrolytes have a low voltage window of about 1.2 V, which is lower than that of organic electrolytes.

Organic electrolytes contain organic solvents and conducting salts that can dissolve together. Organic electrolytes have a high voltage window; thus, devices based on it dominate in the commercial market. Organic electrolytes have a higher voltage range up to 3.5 V compared to aqueous electrolytes. Types of organic electrolytes that are widely used as solvents are acetonitrile and propylene carbonate. Acetonitrile may dissolve large amounts of salts but has environmental and toxic issues, while propylene carbonate is environmentally friendly, conductive, and has a larger electrochemical potential window and high operating temperature. Moreover, tetraethylammonium tetrafluoroborate, tetraethyl phosphonium tetrafluoroborate, and triethyl methylammonium tetrafluoroborate are types of organic salts that have been used in electro-chemical supercapacitor electrolytes. Solubility can be increased by salts with less symmetrical structures due to less crystal-lattice energy [173]. The properties of solvents and salts, such as size of ion, ion–solvent interaction, conductivity, and viscosity, are the main aspects that affect the performance of organic electrolyte-based semiconductor devices. The response to the ionic size of trimethylethyl ammonium BF_4_ and trimethylpropyl ammonium BF_4_ has been observed in supercapacitors; the capacitance using microporous activated carbon electrodes was 10 wt.% higher than tetraethylammonium BF_4_. The research found that capacitance depends not on only anions but on the size of the cations. Capacitance is also related to the correlative radius of bare cations. All these factors are related to the pores of working electrodes. The specific capacitance is not only depending on specific surface area but also on the pore size of the carbon materials. The smaller the size of pore in carbon materials, the larger the specific surface area. Unfortunately, organic ions with a larger size cannot easily enter narrow pores and lower the specific capacitance. Therefore, the size of ions in electrolytes must be comparable with the pore size of the carbon material of the electrode to increase the specific capacitance [176].

### 7.2. Quasi-Solid-State Electrolytes

For the fast growth of flexible electronic devices, quasi-solid-state electrolytes receive more interest. They have lot of advantages compared to other electrolytes, such as good ionic conductivity, leakage-free, and easy packaging. Solid-state electrolytes depend on polymer electrolytes for developing supercapacitor devices. Because of the existence of a liquid form, gel polymer electrolytes (GPEs) are known as quasi-solid-state electrolytes. As mentioned above, GPEs are a host of polymer and aqueous electrolytes or a salt that is dissolved in a solvent. Among all types of solid-state electrolytes, GPEs have the highest ionic conductivity. On the other hand, owing to the presence of water, GPEs may face a problem with low operating temperature and poor mechanical properties. GPEs have many advantages, such as tenable and bendable structures. In order to attain energized, flexible, and wearable supercapacitors with high power and energy densities, gel polymer electrolytes (GPEs) are the most suitable materials. There are many types of GPE, and hydrogel-based aqueous electrolytes have attained more attention in research because of their interesting mechanical properties, versatility, and good ionic conductivity. Gel-polymer electrolytes are basically synthesized using organic solvents. Many matrices of polymers are used to prepare GPEs, including poly (acrylic acid) (PAA), poly (vinyl alcohol) (PVA), potassium polyacrylate (PAAK), and poly (ethyl oxide) (PEO). Examples of organic solvents used are ethylene carbonate (EC), dimethylformamide (DMF), propylene carbonate (PC), and mixtures of both (PC-EC and PC-EC-DMC).

### 7.3. Polymer Hydrogel Electrolytes for Supercapacitors

The replacement of organic solvents with water as a plasticizer in gel-polymer electrolytes reduces the device cost fundamentally [174,175]. This gel-polymer-carrying water electrolyte is known as the hydrogel-polymer electrolyte [177]. Hydrogels have the characteristic ability to conduct ions with dimensional stability, and because of this, many types of energy-storage devices have been fabricated using hydrogels as electrolytes. Supercapacitors and batteries are included. In fact, gel-polymer electrolytes possess higher ionic conductivity compared to solid-state electrolytes because of the liquid phase presence. There are many hosts for polymer which have been explored to build up polymer hydrogel electrolytes. For example, chitosan, sodium alginate, agar, cellulose, starch, poly (ethylene oxide) (PEO), poly (acrylic acid) (PAA), poly (acrylamide), poly (ether ether ketone) (PEEK), and poly (vinyl alcohol) (PVA) [140,178,179,180,181,182,183,184,185,186].

Chen et al. studied six different types of PVA-based hydrogels using different types of electrolytes, namely H_2_SO_4_, KOH, NaOH, NaCl, H_3_PO_4_, and KCl, for supercapacitors with graphene-based electrodes. The results concluded that by comparing all PVA-based hydrogels, the finest capacitive performance is represented by PVA-H_3_PO_4_ hydrogel electrolytes. This comparison was obtained from electrochemical measurements. Compared to other ions in gel electrolytes, which are K^+^, Na^+^, Cl^−^, and OH^−^, the H^+^ ion has the smallest ionic radius, which contributed to the high capacitance of PVA/H_3_PO_4_. The smaller the radius of ions, the faster and easier the ions can diffuse through the electrode. At equivalent molar concentrations, a lot more unbounded ions can be created by H_3_PO_4_ than by NaOH or NaCl. Thus, from all the salt electrolytes studied, PVA-H_3_PO_4_ hydrogel electrolytes show a higher specific capacitance [187].

**Figure 18:**
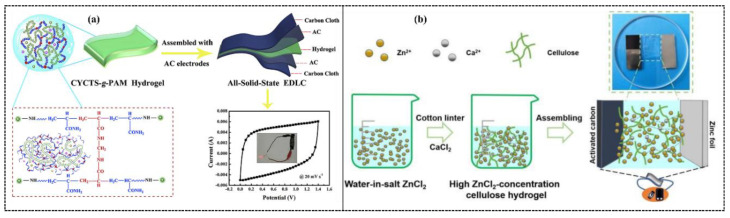
(**a**) Synthesis and supercapacitor assembly containing prepared covalently carboxylated chitosan hydrogel electrolytes [178]. (**b**) Synthesis of physically crosslinked cellulose hydrogel electrolytes and zinc ion hybrid supercapacitor assembly [180].

Chitosan-based hydrogel electrolytes are gaining increasing interest owing to their size-controlled morphology, biodegradability, and biocompatibility. Carboxylated chitosan-g-poly (acrylamide)/lithium sulphate hydrogel electrolytes have been recently prepared via covalent crosslinking by Yang et al. The hydrogel electrolytes exhibited higher ionic conductivity (1.74 × 10^−2^ S/cm) and enhanced mechanical strength. Sandwiched hydrogel electrolytes between carbon electrodes delivered an energy density of 8.7 W h/kg at a power density of 350.3 W/kg in a wide potential window of 0 to 1.4 V. Figure 18a reveals the synthesis and supercapacitor assembly containing the prepared hydrogel electrolytes [178].

Yang et al. reported zinc ion hybrid supercapacitors using biodegradable cellulose-based hydrogel electrolytes containing highly concentrated zinc chloride. The supercapacitors achieved a specific capacitance of 193 mA h g^−1^, an energy density of 192 W h kg^−1^ at a power density of 16,976 W kg^−1^, and the supercapacitor could be stable and worked at −20 °C. Synthesis of the hydrogel electrolytes and the hybrid supercapacitor assembly is shown in Figure 18b [180]. Furthermore, all-in-one supercapacitor fabrication is a new approach, where the electrodes and electrolytes are in very close contact. Biodegradable cellulose hydrogel crosslinked with epichlorohydrin at low temperatures as electrolytes and nitrogen-doped graphene hydrogel as electrodes have been used to fabricate an asymmetric supercapacitor. The supercapacitor revealed an 89.5% rate capability at a current density of 16 A/g and 93.9% capacitance retention after 5000 charge/discharge cycles [188].

Wang and colleagues have reported the chemical crosslinked polyvinyl alcohol hydrogel electrolyte for supercapacitors. Before hydrogel-based electrolytes, separators such as polyethylene and polypropylene faced limited ionic conductivity. The ionic conductivity was 8.2 × 10^−2^ S cm^−1^, which is near to a standard aqueous H_2_SO_4_ electrolyte (~10^−1^ S cm^−1^) [189]. The polymer electrolytes of PEO-KOH-H_2_O exhibited conductivity of ions roughly around 10^−3^ S cm^−1^. An electric double-layer capacitor using the respective electrolytes was fabricated by Lewandowski et al. The capacitance was comparable with the supercapacitor containing liquid electrolytes. Moreover, potassium polyacrylate (PAAK) polymer hydrogel electrolytes were prepared using KOH aqueous solution as a source of ions for application in nickel/metal hydride batteries. The polymer hydrogel electrolyte achieved very high ionic conductivity up to 6 × 10^−1^ S cm^−1^. This amount is almost comparable to KOH aqueous solution. The experiment of polymer hydrogel electrolytes with the Ni/MH cell also exhibited a higher capacity of charge storage with a lower leaking rate of electrolytes compared to the case of aqueous solution for KOH [190]. PVA-based hydrogel electrolytes have been largely used to build flexible supercapacitors because of their low cost, non-toxicity, and chemical stability [12].

### 7.4. Flexible Polymer Hydrogel Electrolytes

Modification and efforts have been made in achieving new hydrogel electrolytes with enhanced mechanical flexibility and ionic conductivity to produce flexible supercapacitors. One of the methods of improving the chemical and mechanical performances of original PVA-based hydrogel electrolytes is by crosslinking with glutaraldehyde (GA). Wang et al. prepared a PVA-H_2_SO_4_ hydrogel film through film casting and polyaniline (PANI) embedded by in situ growing to create a new conceptual supercapacitor. Crosslinked film with PVA showed great elasticity and reached about a 300% value of strain and strong mechanical strength followed by high ionic conductivity compared to the original rigid PVA film. Crosslinked PVA-H_2_SO_4_ film possessed 90% water in the polymeric network and had a high ionic conductivity value of 0.082 S cm^−1^. The fabricated supercapacitor achieved large areal capacitance of 488 mF cm^−2^ and retained 90% of its original capacitance after 7000 cycles [189].

Through a dual-network design, highly elastic hydrogel electrolytes were fabricated by Lopez and colleagues. In this network, covalent crosslinking supplied the elasticity while the stress produced by stretching or bending hydrogen bonds was dissipated by the hydrogen bonds in amide groups. Hydrogel’s ionic conductivity can be magnified with the introduction of ionizable functional groups with polymer networks, usually known as polyelectrolytes, polyampholytes, and zwitterionic networks. Polyelectrolytes can be negatively or positively charged, polyampholytes can be both positively and negatively charged, and zwitterionic is a monomer with both negatively and positively charged groups. The existence of hydrogen-bonding and electrostatic attraction causes the polyampholyte hydrogel to exhibit mechanical stability [191].

**Figure 19:**
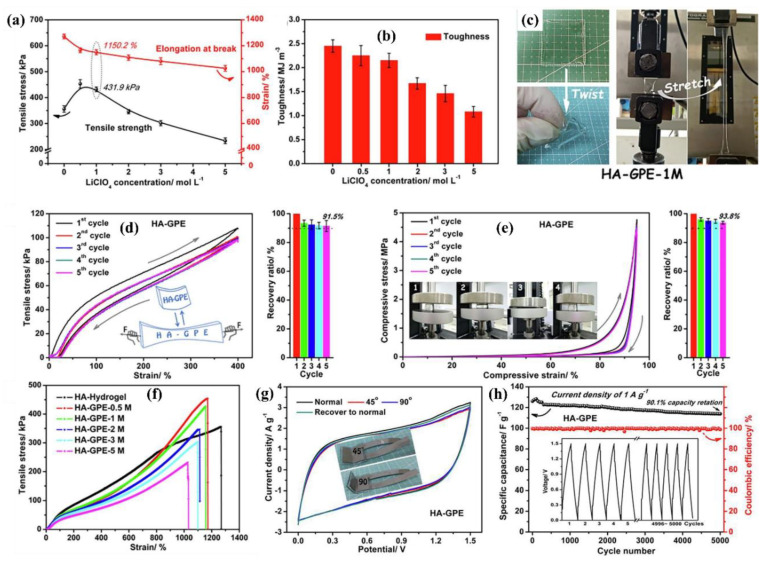
The comparison of (**a**) tensile strength and elongation at break and (**b**) toughness of HA-GPE-x. (**c**) Digital images of HA-GPE-1M with high flexibility (left) and stretchability (right). Cycling (**d**) tensile stress–strain and (**e**) compressive stress–strain curves of HA-GPE at a strain of 400% and 95% and corresponding recovery ratio. (**f**) Tensile stress–strain curves of HA-GPE-x with different LiClO_4_ concentrations. (**g**) Cyclic voltammetry (CV) curves at normal and varied bending angles (45°, 90°). (**h**) Cycling performance collected for the current density of 1 Ag^−1^ (black: capacity retention; red: Coulombic efficiency as a function of cycle number) [192].

Na et al. prepared mechanically robust hydrophobic associated hydrogel electrolytes (HA-GPEs) of acrylamide through UV light irradiation, where LiClO_4_ was incorporated as a source of ions. Replacement of conventional covalent crosslinking with hydrophobic units into hydrophilic chains forms micelles when exposed to an aqueous environment, acting as physical crosslinking. The reversible physical crosslinking points based on hydrophobic association efficiently dissipate the crack energy upon application of a large strain and can recover its bonding upon unloading, thereby rendering upon the hydrophobically-associated hydrogel an unusual mechanical strength and resilience during deformation. The mechanical flexibility of hydrogel electrolytes and their electrochemical performance is shown in Figure 19a–h. It could be observed that mechanical strength was significantly affected by LiClO_4,_ which was due to the proper dispersion of Li^+^ ions and interaction with ethylene oxide present on the hydrophobic moiety, thus promoting the migration of ions. Tensile strength decreased gradually with the increase in concentration of LiClO_4_ and displayed the minimum strength at 5 mol L^−1^. In addition, elongation at break also decreased from 1271.6 to 1026.2% with the increase in concentration of LiClO_4_, which means excess LiClO_4_ was unfavourable for mechanical strength and could have a negative influence on the toughness of the HA-GPEs. Electrochemical results of the supercapacitor exhibited nearly the same results under normal and bending angles and the supercapacitor retained 90.1% capacitance retention and 100% coulombic efficiency after 5000 cycles [192].

**Figure 20:**
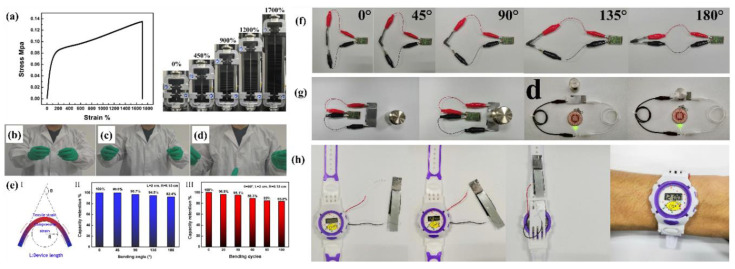
(**a**) Stretching ability of sodium alginate/poly (acrylamide)/poly (acrylic acid)/zinc sulfate (SA-Zn) hydrogel electrolytes. (**b**–**d**) Flexibility demonstration of SA-Zn hydrogel electrolytes. (**e-I**) Schematic of the structure and three key parameters (θ: bending angle, R: bending radius of curvature, and L: length of the device) that are used to demonstrate the bending state of H-ZHS. (**e-II**) Capacity retentions of H-ZHS at different bending angles (0–180°), fixed length (2 cm), and bending radius (0.15 cm). (**e-III**) Capacity retentions of H-ZHS upon 100 bending cycles at fixed bending angle (90°), bending radius (0.15 cm), and length (2 cm). (**f**) Photographs of an electrical watch powered by H-ZHS at different bending angles (0–180°). (**g**) An electrical watch and (**h**) a green LED powered by H-ZHS compressed by a 200-g load. (**h**) Photographs of H-ZHS attached on a wristband to power an electrical watch [179].

Han et al. examined the mechanical strength of the zwitterionic double network sodium alginate/poly (acrylamide)/poly (acrylic acid)/zinc sulfate (SA-Zn) hydrogel electrolytes and the fabricated supercapacitors. The mechanical strength of the hydrogel electrolytes and electrochemical performance of the supercapacitor at different stretching states are displayed in Figure 20a–h. Due to the double network structure, the SA-Zn hydrogel exhibited good mechanical properties and excellent stretchability when elongated to a length of more than 1700% under 0.13 MPa pressure. The excellent stretching flexibility of the SA-Zn hydrogel should be attributed to the dynamic coordination interaction and electrostatic interaction between Zn^2+^ ions and molecular chains in the SA-Zn hydrogel, which are relatively weak but still stronger than hydrogen bonds [184].

A super flexible supercapacitor using polymer hydrogel has been synthesized with poly (acrylamide) (PAM) crosslinked by vinyl hybrid silica nanoparticles (VSNPs). In order to prepare the electrolytes, acrylamide was used as a monomer and the as-prepared VSNPs were polymerized together to form the VSNP-crosslinked PAM hydrogel with ammonium persulfate as the initiator and phosphoric acid as an ionic source. PAM was used to provide hydrogen bonds. Some of them recombined dynamically when they were separated during stretching to aid energy dissipation and network homogenization. VSNPs were used as a tension buffers to dissipate energy when high pressure was applied, while ionic conductivity was due to protons in the hydrogels. The VSNP-PAM polyelectrolyte film was pre-stretched to more than 1000% strain for the manufacturing of the super-stretchable supercapacitor. Many loads were placed on top of the supercapacitor, resulting in the supercapacitor being intrinsically compressible due to the flexibility of the VSNP-PAM electrolytes [11].

### 7.5. Self-Healing Properties of Polymer Hydrogel Electrolyte

If you cut a hydrogel into two halves and then allow it to come back to the normal state, i.e., the two halves automatically bond back together, it is known self-healing, because two pieces bond back together to recover their original shape. So, the autonomous self-healing of hydrogel is basically a unique property which pushes the material to naturally heal the damage and come back to its original/normal state. This autonomous self-healing is usually observed in materials that are prepared beforehand and no interference of any external stimuli is allowed, which is good to increase the healing depth/extent. Synonymously, another term is self-recovery, which points toward the healing of any internal damage and looks for any viscoelastic properties of a material, e.g., molecular diffusion of ionic crosslinks to regain the 3D polymer network structure after rheological deformation [3].

It is beneficial to develop materials that are capable of self-healing damage in the sense that they help in increasing the lifespan and conserving the actual properties of the material. For some applications, it helps in eschewing failures, which in turn multiplies the material’s safety. Compared to natural phenomena, hydrogels and other synthetic materials tend to fail or get damaged with no way of repairing them. So, the natural phenomenon of self-healing is now being included in polymer materials such as hydrogels [193].

Hydrogels are being tremendously used in a variety of applications, such as in flexible energy-storage devices, soft robotics, biomedicine, tissue engineering, and wound dressing. They are also used in hygiene products and contact lenses and in a lot more money-oriented products. Soon before any damage becomes quite noticeable, the cracks have already been caused. However, present polymeric materials fail to prevent any mechanical damages. The only applicable solution to this problem is the evolution and success of self-healing hydrogels. For a hydrogel to be able to self-recover automatically, self-healing properties/tendencies are chemically included in the polymeric structure of the hydrogels by adding reversible crosslinks/bonds. Self-healing hydrogels must stick to a single design and, preferably, it should be synthesized from a non-hazardous material, and it should be able to avoid deteriorating early when used for applications. Simultaneously, it should also be able to recover from damage without using any external stimulus and should heal from the micro to the macro level; most importantly, it should be able to hold on to the original rheological and mechanical properties.

Two main approaches allow us to conduct research on self-healable hydrogels, and those are dynamic covalent bonding, which is chemical crosslinking, and the second is non-covalent bonding, such as physical crosslinking. Hydrogels based on dynamic/chemical crosslinking require no external stimuli but are not very usual. However, physical crosslinking or noncovalent bonding is usually the one used for self-healing hydrogels, either individually or sometimes merged with hydrogen bonding, ionic bonding, supramolecular interactions, hydrophobic bonding, and molecular diffusion and chain entanglement [194].

Self-healing hydrogels consist of non-covalent interactions, which are very weak and reversible in nature, due to which self-healing hydrogels have substandard mechanical properties. However, tough self-hydrogels have been synthesized that can withstand deformation by using different crosslinking mechanisms, such as the ones found in interpenetrating polymer networks (IPNs). IPNs usually contain two, or sometimes more, networks that are twisted together without the presence of any crosslinks. Sometimes, the addition of another strong network results in limiting the self-healing tendencies. For instance, if a non-reversible chemical crosslink is added into the interpenetrating polymer network, it has been observed that its self-healing does not reach 100% recovery. Hence, progress in the synthesis of robust self-healing hydrogels still needs to be realized.

Self-healable supercapacitors are developed in a short lifespan. Self-healable supercapacitors improved their reliability due to the spontaneous recovery of chemical and mechanical properties if there is any physical damage, such as cutting or breakage. Recently, hydrogel electrolytes have received striking attention because of their self-healable property with high ionic conductivity, better mechanical properties, and greater recyclability. Other than a long life, large power density, and quick charge and discharge capability, capacitors which convey a self-healing ability also own better reliability. This is because they tend to automatically recover the mechanical as well as the electrochemical properties when facing physical harm, such as cuts and breakage. Hydrogel electrolytes are prepared using self-mending polymers in order to build self-healing electrochemical capacitors. The moment when the fabricated capacitors are damaged physically, such as with cuts, they immediately reconstruct their configuration. They are restored by hydrogen bonding or magnetism, which is part of reversible physical interactions.

**Figure 21:**
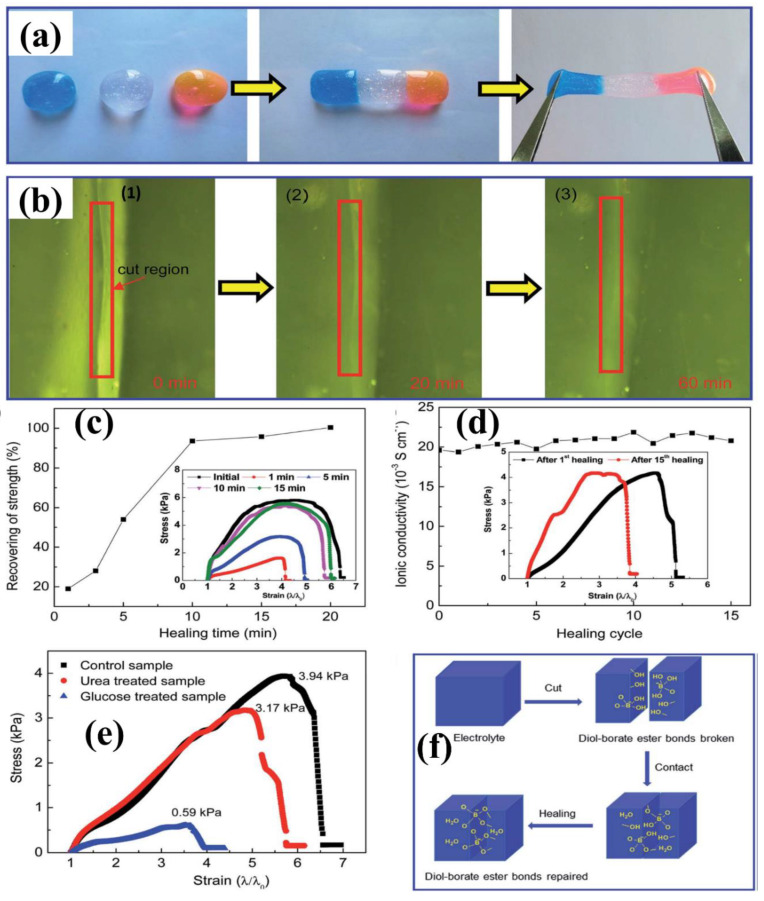
(**a**) Optical images showing self-healing of the PVA-g-PAA/KCl pieces under ambient conditions. (**b**) Fluorescence microscopy images of the electrolytes after healing for (**1**) 0 min, (**2**) 20 min, and (**3**) 60 min. (**c**) Effect of healing time on the mechanical properties and mechanical healing efficiency of the PVA-g-PAA/KCl electrolytes; the grafting amounts of PAA and KCl contents were 3.5 wt.% and 200 mM, respectively. (**d**) Ionic conductivity of the electrolytes after different cutting/healing cycles; the inset shows the mechanical properties after the 1st and 15th cutting/healing cycles. (**e**) Effect of urea and glucose treatments on the mechanical properties of the healed electrolytes. (**f**) Schematic illustration of the self-healing mechanism [195].

Even though all PVA-based electrolytes have hydroxyl functional groups, many hydrogel electrolytes rarely show the behavior of self-healing by hydrogen bonding. Many of them showed low mechanical strength and are brittle. To synthesize electrolytes with self-healable properties, PVA was grafted with poly (acrylic acid) (PAA) and later converted to hydrogel in the presence of KCl and borax due to the formation of dynamic borate-diol ester bonds. The grafting reduced the PVA chain clotting, which was because of the salts, which made the prepared PVA-g-PAA/KCl highly flexible and conductive hydrogel electrolytes. In the absence of any exterior stimulus, perfect self-healing efficiency through dynamic borate ester-bonding was also displayed by the electrolytes. Ester-bonding of dynamic diol-borate is an agreeable plan to process many types of self-healable hydrogel electrolytes due to their good reversibility, easy formation, and electrochemical inertness. Self-healing hydrogel electrolytes have potential applications in smart and flexible energy-storage devices. Figure 21 reveals the optical and microscopic images of self-healing characteristics of hydrogel electrolytes, recovery times, effects on ionic conductivity, stress versus strain, and a schematic illustration of the self-healing mechanism [195].

**Figure 22:**
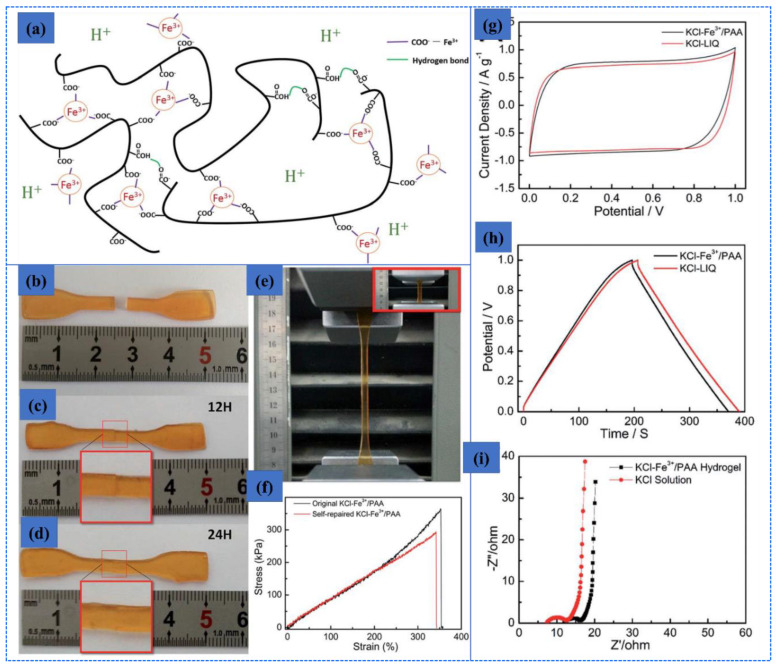
(**a**) Schematic illustration of the internal crosslinking effect of the KCl–Fe^3+^/PAA hydrogel. (**b**–**d**) Self-healing of a dumbbell-shaped hydrogel at room temperature: (**b**) the hydrogel after being cut, (**c**) the hydrogel after healing for 12 h, and (**d**) the hydrogel after healing for 24 h. (**e**) Stretching the self-healed hydrogel up to 200%. (**f**) Comparison of stress–strain curves between the original and self-repaired KCl–Fe^3+^/PAA hydrogel. (**g**) CV curves of the KCl–Fe^3+^/PAA F-supercapacitor and KCl L-supercapacitor at a scan rate of 5 mV s^−1^. (**h**) Comparison of the charge/discharge profile between the KCl–Fe^3+^/PAA F-supercapacitor and KCl L-supercapacitor at a current density of 0.5 A g^−1^. (**i**) Comparison of the Nyquist impedance plot of the KCl–Fe^3+^/PAA F-supercapacitor and KCl L-supercapacitor [196].

Non-covalent physical interactions, such as hydrogen bonding and host–guest and ionic interactions, in the hydrogels introduce unique reversibility and fascinating self-healability. Poly (acrylic acid) containing a carboxyl group with the ability to develop such interactions with other groups and metal ions would be able to form reversible and self-healing hydrogels. One example is the formation of Fe^3+^ ion-crosslinked highly poly (acrylic acid) hydrogel electrolytes. The flexibility, reversibility, and self-healability of the hydrogel electrolytes came from the ionic interaction between carboxylate ions and Fe^3+^ ions (Figure 22a–e). The hydrogel electrolytes maintained good mechanical performance (extensibility > 700%, and stress > 400 kPa) and excellent conductivity (0.09 S cm^−1^), which completely satisfy the demands of flexible supercapacitors. After being assembled with graphene foam-supported polypyrrole electrodes, the electrochemical performance of this self-healable supercapacitor was comparable to that of its liquid electrolyte counterpart (Figure 22f–h) [196].

**Figure 23:**
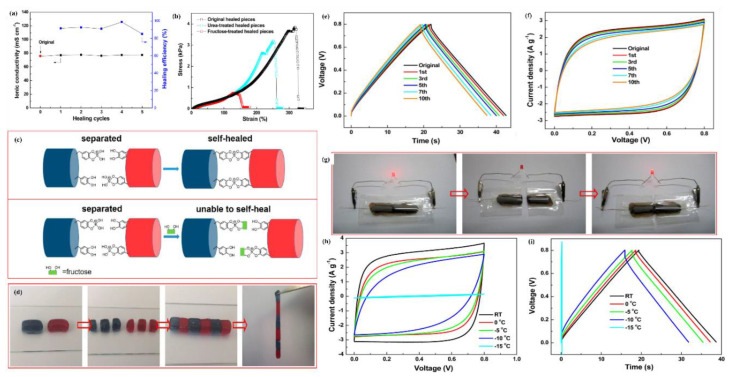
(**a**) Mechanical healing efficiency and ionic conductivity of the electrolyte after multiple cut/healing cycles. (**b**) Effect of urea and fructose treatments on the mechanical properties of the healed pieces. (**c**) Illustration of the self-healing mechanism of the electrolyte. (**d**) Optical images of self-healed hydrogel electrolyte. (**e**) GCD profiles at 1.0 A g^–1^ (**f**) CVs at 100 mV s^–1^, (**g**) Four capacitors were connected in series to light up LED bulb through cut/healing operations. (**h**) Electrochemical performances of the capacitor at low temperature, CVs at 100 mV s^–1^, (**i**) GCD profiles at 1.0 A g^–1^ [197].

Self-healing supercapacitors usually comprise aqueous hydrogel polymer electrolytes containing acid or salt as source of ions. A significant drawback of aqueous hydrogel electrolytes is freezing at low temperatures that deteriorates their ionic mobility, flexibility, stretchability, and self-healability. However, addition of large amounts of salt can damage the three-dimensional network of hydrogel that could alter the flexibility and intrinsic self-healability. Until now, it has been a great challenge to add large amounts of salt without damaging the network of a hydrogel. Therefore, self-healable, cold-resistant sodium alginate hydrogel electrolytes physically crosslinked via catechol borate ester bonding are vital and can maintain self-healing (up to 10 cycles), and fabricated supercapacitors can retain efficiency (80% capacitance) at a low temperature of −10 °C. The self-healability came from dynamic catechol ester bonding and KCl was the source of ions. However, upon addition of fructose, the self-healing and mechanical properties changed owing to the formation of new ester bonding between borate and OH group of fructose. Figure 23a–i describes a comprehensive overview on the self-healing mechanism, self-healing efficiency, mechanical strength, and electrochemical performance of self-healed supercapacitors as well as at low temperature [197].

**Figure 24:**
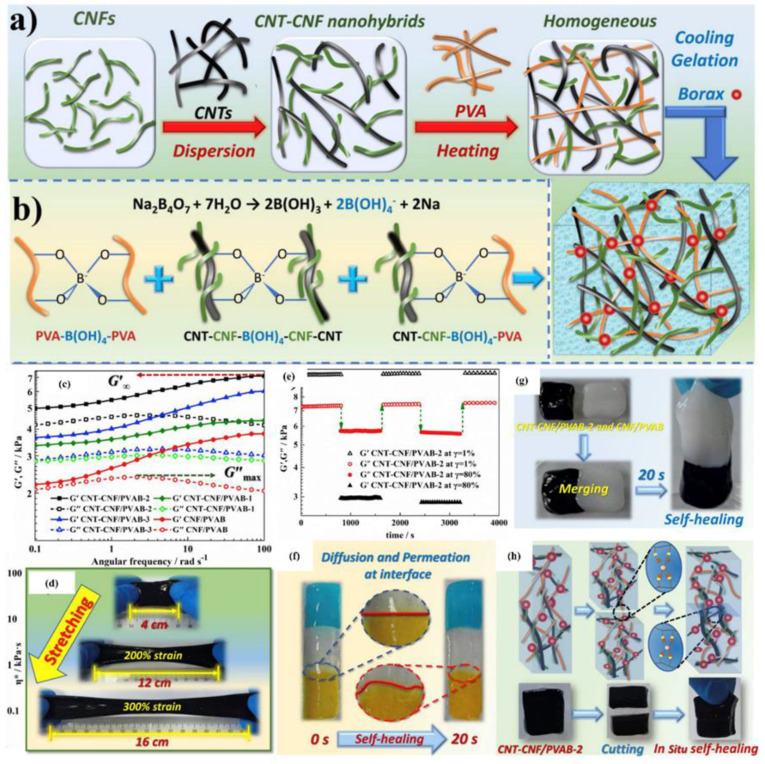
(**a**) Fabrication process of CNT-CNF/PVAB composite gels. (**b**) Multi-complexation between CNT-CNF nanohybrids and PVA dynamically cross-linked by borax. (**c**) G’ and G″ curves as a function of ω. (**c**) η* and G* versus angular frequency ranging from 0.1 to 100 rad s−1. (**d**) CNT-CNF/PVAB-2 hydrogel being stretched to more than 300% of the initial length. (**e**) The G″ and G′ versus time in continuous step strain of CNT-CNF/PVAB-2 composite gel. (**f**) Illustration of CNT-CNF/PVAB and CNF/PVAB hydrogels being completely merged. (**g**) Illustration of self-healing behavior for CNF/PVAB and the interfacial permeation during seal-healing. (**h**) Illustration of healing in situ for CNT-CNF/PVAB-2 gel and self-healing mechanism of composite gels [203].

Numerous ways have been adopted to prepare self-healable hydrogel electrolytes, such as hydrogen bonding, ionic interactions, polyion-complexation, polyampholyte, and hydrophobic association [198,199,200,201]. Natural polymers, synthetic polymers, and both types of polymer combine via physical interactions to form hydrogel electrolytes. Modified chitosan and poly (acrylic acid) can interact ionically to form tough and self-healable hydrogel electrolytes. Similarly, polyion-complex hydrogel electrolytes have been reported with dynamic ionic interactions having strong mechanical strength, resistant to fatigue, and self-healable properties [200]. Peng et al. synthesized self-healable hydrogel electrolytes of PVA/graphene oxide crosslinked via borax containing KCl as a source of ion. The prepared hydrogel electrolytes could exhibit outstanding self-healing properties and repaired within 5 min after rejoining the pieces. The fabricated supercapacitors also delivered a specific capacitance of 156 F/g at 0.3 A/g and could store the electrochemical performance even after the seventh cycle [202]. Han et al. fabricated a self-healable and flexible supercapacitor using hydrogel electrode and hydrogel electrolytes. Hydrogel electrode was prepared using carbon nanotubes (CNT), cellulose nanofibers (CNF), and PVA, while the hydrogel electrolytes were CNF/PVAB crosslinked with borax. In the hydrogel electrode, the conductivity of CNTs and template function of cellulose nanofibers was combined. Cellulose also helped the uniform dispersion of CNT in PVA hydrogel to form free-standing composite hydrogels. PVA hydrogel electrolytes were prepared by borax crosslinking. The prepared hydrogels were self-healable and flexible. The CNT/CNF/PVAB hydrogel was mechanically strong compared to the CNF/PVA hydrogel electrolytes and the compression and storage modulus of CNT/CNF/PVAB were 2.7- and 1.9-fold greater than CNF/PVAB hydrogel electrolytes, respectively. The higher compression and storage modulus were due to the presence of CNTs. The plausible self-healing can be observed from the synthesis mechanism where inter- and intra-molecular hydrogen bonding and covalent linkages between OH of PVA and borax or CNT/CNF nanohybrids take place. In addition, the self-healing was very fast, and it took 0.33 s. During this time, the dynamic linkages disintegrated and re-established. The fabricated supercapacitor achieved a specific capacitance of 117.1 F/g and a capacitance retention of 96.4% after 1000 cycles. The self-healable and flexible supercapacitor demonstrated an ideal capacitance retention (98.2%) after ten damaging/self-healing cycles and capacitance retention (95%) after 1000 cycles under various deformation. Figure 24 reveals the synthesis scheme, mechanism, mechanical properties, and self-healability of the hydrogel electrode and electrolytes [203].

**Figure 25:**
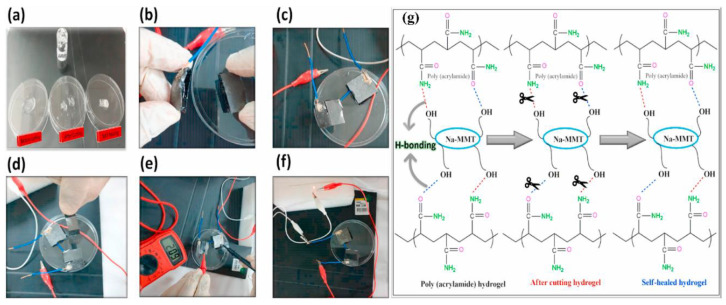
(**a**) AAM3 hydrogel electrolytes before cutting, after cutting, and self-healed; (**b**) supercapacitor after cutting into two pieces; (**c**) rejoining of cut pieces; (**d**) graphite electrode broken ends connected using carbon tape; (**e**) voltage stored after charging; (**f**) discharging of two supercapacitors connected in series through LED. (**g**) Structural illustration of self-healing mechanism in poly (acrylamide) hydrogel [41].

Hina et al. recently reported self-healable physically clay-crosslinked poly (acrylamide) hydrogel electrolytes and used them in self-healable supercapacitors. Self-healable properties of the hydrogel’s electrolytes are present because of the hydrogen bonding between the NH_2_ group of poly (acrylamide) and the hydroxyl group of clay and ionic interactions between the C=O and Li^+^ ions of lithium trifluoromethane sulfonate. A self-healable hydrogel-based flexible supercapacitor lit up an LED and further verified the real-time application of self-healable hydrogel electrolytes (Figure 25) [41].

#### Evaluation and Mechanism of Self-Healing Ability of Hydrogel Electrolytes

As mentioned earlier, hydrogels that can self-heal without needing any external stimulus are termed as autonomous self-healing hydrogels. The self-healing tendency of hydrogels can be easily evaluated by monitoring their behaviour. For example, one could break a hydrogel in two halves and place them back together to monitor the macroscopic behaviour of healing. There is another way of evaluating the self-healing behavior of hydrogel—check whether it can bear its own weight or not without breaking again. However, there are many other methods to analyse the self-healing ability, such as compression and tension tests carried out to estimate bulk damage. The self-healing efficiency of hydrogels can be determined by the elongation ratio of the pristine (λ_b,0_) and healed (λ_b_) hydrogels using Equation (11) [3].

(11) $$\varepsilon_{H}=\left(\frac{\lambda_{b}}{\lambda_{b,0}}\right)\;\times 100$$

In addition, different efficiency tests are conducted to examine the mechanical strength of hydrogels—for instance, hydrogels that are not mechanically robust are subjected to compression testing since they are unable to bear the tensile test. Another factor that limits self-healing efficiency is usually the type of crosslinking that exists between the bonds. For example, bonds sharing a physical crosslinking can attain up to 100% self-recovery. Although, those hydrogel bonds mean that interpenetrating polymer networks are unable to reach up to 100% self-healing capacity since they have chemical and physical bonding.

Generally, the mechanism of self-healing hydrogels depends upon the reversibility of the crosslinks. Since the number of crosslinks and mechanical robustness of the constituents is counted in the degree of stability and mechanical strength of the hydrogels, dynamic covalent bonding, supramolecular interactions, hydrogen bonding, and ionic and hydrophobic relationships have been included in self-healable polymer hydrogels.

As compared to ionic and dynamic covalent crosslinks, hydrogen bonding is weaker. Partial negative and positive charges occur on both atoms when a hydrogen atom pairs up with an electronegative atom. So, this electrostatic interaction between the two atoms causes the formation of reversible crosslinks between multiple polymer chains. However, usually, it takes less than an hour for the hydrogels to self-heal due to the development of intertwined hydrogen bonds, which leads to readjustment of all the interactions. When the sample is manipulated, there is a reduction in the robustness of the sample hydrogel. This happens in between the self-healing process due to the possible loss of water molecules as required.

### 7.6. Polymer Hydrogel Electrolytes for Batteries

**Figure 26:**
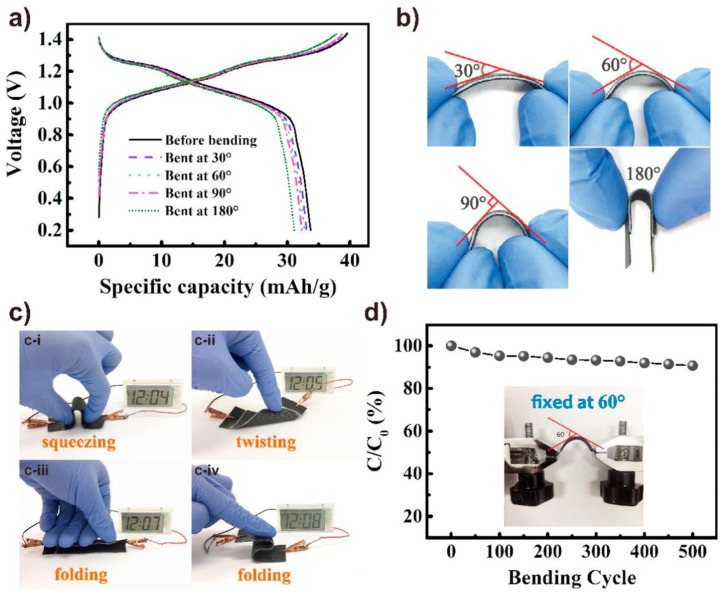
Electrochemical performance of the flexible lithium ion battery under various deformation conditions. (**a**) Galvanostatic charge/discharge under different bending angles. (**b**) Demonstration of bending at different angles. (**c**) Powering an electronic watch when being (**c-i**) squeezed, (**c-ii**) twisted, and (**c-iii**,**c-iv**) folded. (**d**) Capacity retention of the battery after 500 bending cycles at a bending angle of 60° (C_0_ and C correspond to the specific capacity before and after bending, respectively) [205].

Lithium (Li)-, sodium (Na)-, aluminium (Al)-, and zinc (Zn)-based batteries possess high energy density and large operating voltage. Therefore, batteries have a wide potential to power up electronic devices and transportation. These batteries contain different types of electrode materials as well as different electrolytes, such as aqueous, organic, and quasi-solid. The batteries comprised of quasi-solid electrolytes are flexible and can be used in smart and wearable electronics. More specifically, hydrogel electrolytes are water-based systems that reduce the cost of the batteries, improve the ionic and electronic conductivity, and provide solid flexible interphase. Recently, significant development has been made in the fabrication of flexible batteries. Rechargeable lithium ion batteries attained striking attention in energy storage and portable electronics owing to the low reduction potential, high reactivity, and greater capacity. Lithium ion batteries using polymer hydrogel electrolytes exhibited comprehensive performance in terms of security, ion transportation, cost, and capacity. Cellulose-based hydrogel electrolytes are robust in mechanical strength, have high ionic conductivity, support smooth ionic transportation, and show minimal interfacial transportation, outstanding electrochemical performance, and good compatibility with lithium electrode [204]. Hydrogel electrolytes have the capability to retain aqueous electrolytes and separate the two electrodes. Other polymer hydrogels such as PVA hydrogel have also been used as electrolytes in lithium ion batteries. Liu et al. fabricated a lithium ion battery using PVA hydrogel electrolytes and lithium electrode coated with polypyrrole. The coating of polypyrrole was shown to increase the potential window. The battery displayed a 98.7% and 79.8% capacitance retention after 100 and 500 cycles, respectively. The battery also retained its performance under different mechanical deformations, such as bending, twisting, squeezing, and folding. The performance of the battery under different bending angles is shown in Figure 26 [205]. Hydrogel electrolytes have numerous advantages over conventional liquid electrolytes and the batteries are separator free, which can retain a large amount of aqueous electrolytes, have higher ionic conductivity, and have strong interfacial contact with the electrodes. Sodium ion batteries are promising as an alternative of lithium ion batteries because of their greater safety and environmental friendliness. Flexible poly (acrylamide) hydrogel electrolytes are currently used in sodium ion batteries. Herein, organic alloxazine (ALO) is encapsulated in carbon material CMK-3 as an anode and sodium material as a cathode. The anode material exhibited a capacity of 160 mA h g^−1^. Poly (acrylamide) hydrogel electrolytes could protect the dissolution of alloxazine which led to an increase in the cyclic stability with a capacitance retention of 90% after 100 cycles. The assembled battery exhibited a rate of capability of 146 mA h g^−1^, a discharge capacity of 149 mA h g^−1^, and an energy density of 50 W h kg^−1^ at 10 C current density. The batteries could deliver a discharge voltage of 1.03 V. In addition, the poly (acrylamide) hydrogel electrolytes containing sodium trifluoromethane sulfonate retained large water contents and a high ionic conductivity of 1.18 mS cm^−1^ [206].

**Figure 27:**
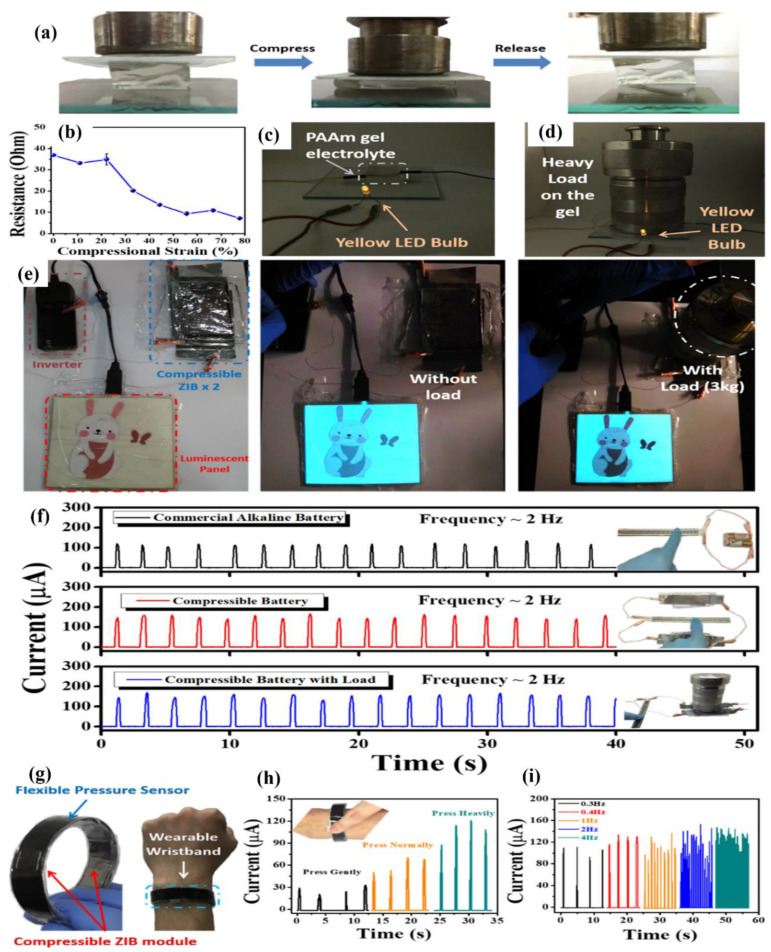
Compressibility of the poly (acrylamide) hydrogel electrolyte (PAAm). (**a**) Images showing the elasticity of hydrogel under compressional and relaxed states; (**b**) resistance values of the poly (acrylamide) hydrogel electrolyte under different compressional strain values from 0 to 77.8%; (**c**,**d**) pictures showing the conductivity of hydrogel electrolyte under relaxed and compressional states with the ability to light a yellow light-emitting diode (LED) bulb; (**e**) optical images showing that two rechargeable Zn–MnO_2_ batteries with poly (acrylamide) hydrogel electrolytes could be used for powering a luminescent panel under normal condition and with a 3-kg load on it; (**f**) comparison between the signals generated by the flexible sensor powered by the commercially available alkaline batteries and our compressible batteries without and with q load on top of it; (**g**) flexible smart wristband integrated from two ZIB modules and a flexible pressure sensor; (**h**) sensory signals of the smart wristband generated by human finger touch under different pressures on the device; (**i**) sensory signals of the smart wristband generated at different frequencies, from 0.3 to 4 Hz, by human finger touch [208].

**Figure 28:**
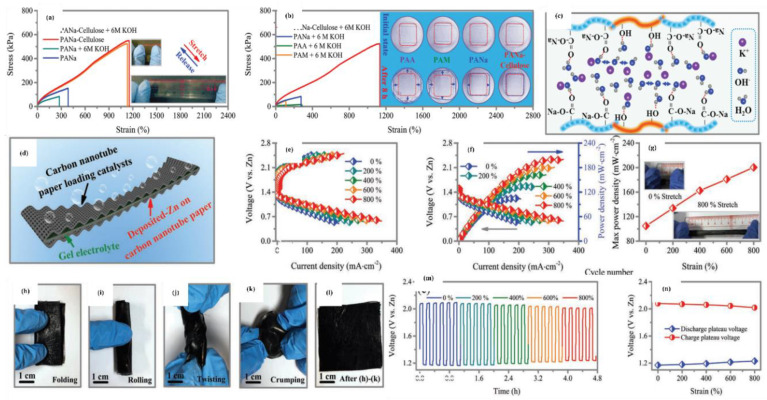
(**a**) Tensile stress–strain curves of the as-synthesized PANa and PANa-cellulose hydrogel electrolytes with and without 300% 6 M KOH + 0.2 M Zn (CH_3_COO)_2_ intake. The insets are optical photos of the relaxed and elongated states of the 300% 6 M KOH + 0.2 M Zn (CH_3_COO)_2_ solution-incorporated PANa-cellulose hydrogel electrolytes showing excellent stretchability; (**b**) comparison of tensile properties of poly (acrylic acid) PAA, poly (acrylamide) PAM, sodium polyacrylate, PANa and PANa-cellulose hydrogel under alkaline condition. The inset is the photos of PAA, PAM, PANa and PANa-cellulose hydrogel at initial state and containing 300% 6M KOH solution for 8 h. The red and blue rectangles represent the shape of hydrogel before and after infiltrating alkaline solution, respectively; (**c**) schematic diagram reflecting structure of PANa-cellulose hydrogel electrolyte entrapped KOH and water via the interactions of hydrogen bonds; (**d**) schematic illustration of 800% stretchable flat-shape zinc–air battery; (**e**) polarization curves; (**f**) corresponding power density curves of the flat-shape highly stretchable zinc–air battery with a strain from 0 to 800%; (**g**) maximum power density as a function of the tensile strain. The insets are the photographs of the flat-shaped zinc–air battery at a fully released state and 800% strain; (**h**–**k**) flat-shape zinc–air battery is subjected to different mechanical deformations sequentially and (**l**) released; (**m**) galvanostatic discharge–charge cycling curves at a current density of 5 mA·cm^−2^ and (**n**) corresponding discharging-charging voltage plateau at different stretching strains [210].

So far, most of the literature has been reported on hydrogel electrolytes in zinc batteries. The use of zinc batteries is owing to the fast ion transportation and dimensional stability. Secondly, zinc as an anode is more promising in batteries due to the numerous advantages, such as lower redox potential, theoretically high capacity of 820 mA h g^−1^, low cost, and environmentally friendly. However, there are a few demerits of zinc material as an anode in the batteries. Counter parts experience slow development, corrosion due to the aqueous environment, parasitic reactions that result in self discharge, and short circuiting. Therefore, flexible semisolid electrolytes are stable and durable. Han et al. reported zinc batteries using gelatin hydrogel electrolytes. Gelatin hydrogel electrolytes deliver benefits due to the zinc battery assembly and its performance. Hydrogel electrolytes were prepared via a simple and cost-effective approach where they can thermo-reversibly gelate and melt above and below the gelation temperature of 38.4 °C. Gelatin molecules formed random coils above the glass transition temperature and locally aggregated to form helical upon cooling. Furthermore, helices could coil into fibers and, finally, into a solid network. On the other hand, gelatin powder dissolved in the aqueous electrolytes on heating and facilitated coating as hydrogel electrolytes on the electrode. Gelatin solidified on cooling and was converted into film, providing mechanical strength to reduce zinc dendrite formation. The assembled zinc battery using gelatin hydrogel electrolyte exhibited a specific capacity of 110.2 mA h g^−1^ [207]. Wang et al. fabricated a zinc battery using poly (acrylamide) hydrogel electrolytes and Zn-MnO_2_ electrode. The fabricated battery displayed excellent performance and tolerance against compression without sacrificing the energy storage capability. The ionic conductivity increased upon compression while the electrochemical performance remained stable. The assembled battery using hydrogel electrolyte powered up a luminescent panel even with a 3-kg weight. In addition, a flexible sensor powered by the flexible battery showed the same response as a commercial alkaline battery. A smart wristband developed using the flexible battery and flexible piezoresistive sensor was powered and monitored the pressure exerted. It is a sign of the potential power source in the smart and wearable electronics. The results of mechanical strength of hydrogel electrolyte and electrochemical performance of the flexible battery and sensor system are presented in Figure 27 [208]. Li et al. reported poly (acrylamide) grafted with gelatin hydrogel electrolytes for a zinc ion flexible battery. The hydrogel electrolytes were in a thin film with a thickness of 30 µm. The resulting hydrogel electrolytes had greater ionic conductivity of 1.76 × 10^−3^ S/cm and excellent water retention capability due to their high hydrophilicity [209]. Sodium poly (acrylate) hydrogel electrolytes have been recently reported for a zinc ion battery. The hydrogel electrolytes showed super absorbency due to the presence of carboxylate ions. There was an electrostatic interaction between carboxylate ions from acrylate group of sodium poly (acrylate) and zinc ions to support ion transportation. This interaction inhibited the formation of zinc dendrites that improved the cyclic stability [12]. Ma et al. reported a zinc-air battery based on alkaline-tolerant dual crosslinked network hydrogel electrolyte of sodium polyacrylate (PANa). Sodium polyacrylate hydrogel electrolyte was prepared in an alkaline medium. Usually, the hydrogels lose stretchability upon preparation in alkaline solution. Herein, the zinc-air batteries were super-stretchable (800% stretchable) in flat and 500% stretchable in fiber mode. Alkaline solution made the hydrogel domains very soft and the carboxylic group was neutralized. The super-stretchable battery exhibited a power density of 108.6 mW cm^−2^ that was enhanced to 210.5 mW cm^−2^ upon stretching to 800%. The mechanical strength of hydrogel electrolytes in flat and fiber modes and the electrochemical performance is shown in Figure 28 [210].

Leng et al. prepared self-healable zwitterionic hydrogel electrolytes for zinc metal batteries. Hydrogel electrolytes attained the highest ionic conductivity of 32.0 mS cm^−1^. The charged groups of the zwitterionic hydrogel reduced the side reactions and homogenized the ionic distribution and accomplished uniform zinc deposition. A long cycling life of 3500 h was attained. In addition, the batteries showed consistent performance under extreme conditions, such as cutting, self-healing, soaking, hammering, washing, burning, and freezing [211]. Multivalent ion batteries have been introduced recently to improve the performance of batteries and to overcome the limited potential window due to the presence of hydrogel electrolytes and can work below the freezing temperature of water. Chen et al. introduced a Zn-MnO_2_ battery and used borax-crosslinked PVA/glycerol hydrogel electrolytes that worked at −35 °C. The presence of glycerol could strongly interact with the PVA chains and inhibited the formation of ice crystals within the hydrogel network. The battery achieved an energy density of 46.8 mWh cm^−3^ at 25 °C and of 25.8 mWh cm^−3^ at −35 °C [212]. Other multivalent ions batteries that could work at different temperatures using different hydrogel electrolytes have been reported to improve the potential window [213,214,215]. Other than this, Al ion batteries have been presented, using flexible and stretchable fiber-shaped hydrogel electrolytes. The results revealed the good mechanical performance of the hydrogel electrolytes in fiber-shape [216]. Flexible and self-healable batteries have numerous applications [217]. Recently, zinc ion batteries fabricated using xanthan gum-poly (acrylamide) hydrogel electrolytes were exploited in submarine applications [218].

### 7.7. Polymer Hydrogel Electrolytes for Energy Conversion Devices

Liquid electrolytes are commonly used in energy conversion devices. However, there are certain limitations of liquid electrolytes as energy storage devices. Therefore, quasi-solid hydrogel polymer electrolytes are a good choice due to their higher ionic conductivity, excellent thermal stability, high mechanical flexibility, sealing, and prominent penetration ability into crystalline porous TiO_2_. Liquid electrolytes are trapped in three-dimensional networks to form homogeneous quasi-solid-state hydrogel electrolytes and I^-^/I_3_^-^ is usually used as a redox couple. Much less work has been reported so far on the use of hydrogel electrolytes for dye-sensitized solar cells (DSSCs).

Conductive hydrogels with fine electronic properties are potential candidates. Das et al. prepared hydrogel of 5,5′-(1,3,5,7-tetraoxopyrrolo [3–4-f] isoindole-2,6-diyl) diisophthalic acid (P) as an organic gelator. The electronic conduction and mechanical strength were improved by adding graphene oxide and PEDOT: PSS, where they interacted with the organic gelator through hydrogen bonding and π–π interaction. The hydrogel was dried and immersed in a ruthenium complex dye (N719). The dye-absorbed hydrogel was air dried and then again immersed in KI/I_2_ solution. The results revealed a 4–5-fold increase in conductivity after adding graphene oxide and PEDOT: PSS. Hydrogels displayed photo-response behavior and a stable photocurrent during on–off cycles. The DSSCs were fabricated using hybrid hydrogel electrolytes that showed 4.5% power conversion efficiency [219]. The investigation of innovative electrolytes based on biodegradable, non-toxic and non-flammable solvents is an up-to-date, intriguing challenge to push forward the environmental sustainability of dye-sensitized solar cells (DSSCs). Miranda et al. reported xanthan gum-based hydrogel electrolytes and I^−^/I_3_^−^ added as a redox couple. Firstly, they designed a very interesting set of experiments varying the different parameters using MODDE software. After careful designing of experiments, a few selected experiments were conducted. The fabricated DSSCs showed a power conversion efficiency of 2.7% and the efficiency was dependent on the dipping conditions and redox mediator concentration [220].

**Figure 29:**
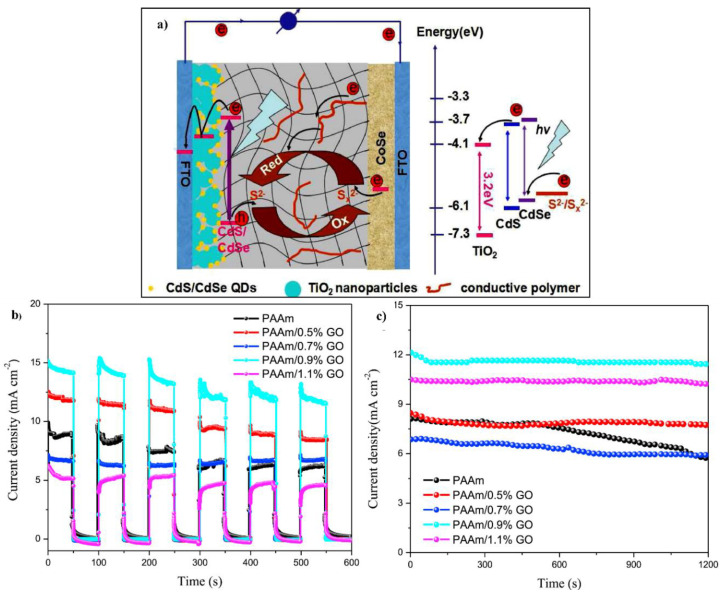
(**a**) The schematic diagram of a quasi-solid-state quantum dot-sensitized solar cell (QDSSC) device from a CdS/CdSe-sensitized TiO_2_ photoanode and conducting hydrogel electrolytes [222]. (**b**) On–off switches and (**c**) photocurrent stability of the gel electrolyte-tailored QDSSCs [223].

Numerous sensitizers have been used in sensitized solar cells (SSCs). However, quantum dots are recognized as highly reliable alternative sensitizers to harvest visible light efficiency. Cadmium sulfide (CdS) and cadmium selenide (CdSe) are frequently used quantum dots. These DSSCs are known as quantum dot sensitized solar cells (QDSSCs). Quasi-solid-state hydrogel electrolytes have shown their best use as stable and potential redox mediators in QDSSCs. Zhexun et al. introduced poly (acrylamide) hydrogel electrolytes for QDSSC. Poly (acrylamide) hydrogel electrolytes were prepared using a covalent crosslinking agent. The hydrogel was dried and immersed in 1 M sodium sulfide (Na_2_S) and 1 M sulphur to prepare hydrogel electrolytes. QD-co-sensitized TiO_2_ was prepared by chemical bath deposition, where CdS and CdSe were deposited on the porous TiO_2_. QD-co-sensitized solar cell was fabricated by sandwiching the hydrogel electrolytes between the QD-sensitized TiO_2_ and copper sulfide counter electrodes. The QD-sensitized solar cell achieved 0.093 S cm^−1^ and a power conversion efficiency of 4.0%, and hydrogel electrolytes effectively hindered the leakage and volatilization of water [221]. Yang et al. introduced a poly (acrylamide) hydrogel along with electronic conductor polypyrrole, polyaniline, and polythiophene. The resulting QDSSC displayed a maximum power conversion efficiency of 2.33% in the case of the poly (acrylamide)/polyaniline hydrogel. The schematic diagram of the QDSSC is shown in Figure 29a [222]. Jin et al. prepared an electron-conducting three-dimensional chemically crosslinked poly (acrylamide)/graphene oxide hydrogel electrolyte for QDSSC. The electron-conducting pathways could conduct electrons from the counter electrode to hydrogel electrolytes and expanded the catalytic area and reduced the charge transfer length. The fabricated QDSSC achieved a power conversion efficiency of 4.10% and attained long-term stability. The results of the stability study are shown in Figure 29b,c. The on–off ability for six cycles and long-term stability of the device were observed. An abrupt increase in current density under simulated sunlight and a quick start up were observed. In addition, stable current density also corroborated the durability. On the other hand, the stability study showed a reduction in stability after 10 min of irradiation in the case of the pure poly (acrylamide) hydrogel electrolytes but continued stability with conducting poly (acrylamide)/graphene oxide hydrogel electrolytes [223].

Chen et al. utilized the natural polymer dextran to prepare physically crosslinked hydrogel electrolytes for a QDSSC as its thermal-reversible properties increased the long-term practical use of the device. It formed a liquid state at high temperatures and allowed better contact between electrodes and then solidified at room temperature in gel form. The QDSSC attained a 4.58% power conversion efficiency under 0.12 sun [224]. An organic gelator has also been used to prepare hydrogels for QDSSCs. The 12-hydroxystearic acid organic gelator gelated the polysulfide liquid electrolyte to polysulfide hydrogel electrolytes. These hydrogel electrolytes changed the transport channel of redox species and accelerated electron recombination at the photoanode/electrolyte interface. The hydrogel electrolytes reduced the power conversion efficiency compared to liquid electrolytes. However, they increased the stability of the QDSSC [225]. Overall, the QDSSC containing dextran hydrogel electrolytes exhibited a higher conversion efficiency compared to the poly (acrylamide) hydrogel electrolyte-based QDSSC and the 12-hydroxystearic acid organic gelator-based QDSSC.

## 8. Conclusions and Future Directions

This review compiles the fundamental concepts of hydrogels, such as materials involved, synthesis approach, classification of hydrogels, ways of crosslinking, types of bonding, and, most importantly, the properties of hydrogels for abundant applications. The unprecedented properties and applications of hydrogels are highly dependent on the selection of polymers and crosslinking agent, way of synthesis, and bonding involved. Ionically conductive hydrogels as electrolytes with outstanding performances in electrochemical devices are swiftly growing in number. The flexibility and mechanical strength of hydrogel electrolytes have also been elaborated comprehensively. To power up wearable electronics, smart, light-weight, and flexible components are the most promising option. However, the flexible devices need to be mechanically sustainable and robust. Therefore, intrinsically flexible ionically conductive hydrogels are ideal frameworks to design required energy storage and conversion devices, as hydrogels can be sustained under large mechanical strain and can hold greater water contents in three-dimensional networks that provide enormously wide surface areas for electrochemical reactions. In addition, self-healing properties of hydrogels are the other significant requirement of the modern era. Herein, the progress related to conductive self-healing hydrogels has been summarized. The self-healing mechanism has been demonstrated significantly in detail.

Potential development has been made in hydrogels in the last few years. However, further understanding regarding rational regulations of the structure and properties of the hydrogels are required to meet the different demands in daily life.

1. With the passage of time, the synthesis and application of hydrogels is becoming more complex day by day. Hydrogels exposed certain limitations, such as fatigue and cracks under long-term load upon exposure in extreme environmental conditions. These limitations are attributed to their chemical composition and topology of hydrogel network. These limitations can be overcome by forming double crosslinked networks.
2. Low conductivity sometimes become an unanticipated challenge in the real-time application of hydrogels. The conductivity, mechanical strength, and optoelectronic properties of hydrogels can be reinforced by incorporating conducting materials, such as nanofillers, to form hybrid hydrogel networks owing to the synergistic interaction between components and the larger surface area of the nanofillers. Nanofillers used to improve conductivity are carbon nanotubes, carbon dots, graphene, metal oxide/sulfide/phosphate, and conducting polymers.
3. According to the percolation theory, flexible electrochemical devices can be developed by integrating hydrogels with certain electroactive materials, such as nanofillers. However, the balance between electroactive materials and hydrogels must be considered to attain flexible devices. In addition, more specifically, nanofillers improve the cycling stability, ionic diffusion, and connectivity of channels in the porous networks of hydrogel electrolytes.
4. Hydrogels with unique properties will be appropriate candidates for electronic, sensing, cell imaging, drug-delivery, and tissue-engineering applications. Highly conducting hybrid hydrogels can be extended to produce artificial skin, soft robotics, tissue generation, 3D printing, etc.
5. Ionically conductive hydrogels are applicable for limited bioelectronics. Advanced fabrication methods can introduce hydrogels in bioelectronics at a large scale and significantly support their success. Three-dimensional printing and ink-jet printing methods of ionic hydrogels are quite new developments and can be promising directions for future developments.

## Figures and Tables

**Table 1:** Crosslinking of polymers through small molecules.

	Agent	Target Functional Group	Reaction Conditions	Crosslinkage	Comments
Small molecules	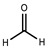 Formaldehyde	Primary amines and aldehydes	Reaction favors basic and neutral pH	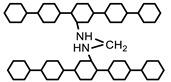	Reaction takes one hour to complete. Difficult to remove trace formaldehyde.
	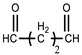 Glutaraldehyde	Primary amines and aldehydes	Reaction favors basic and neutral pH	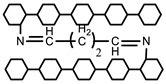	Reaction takes one hour to complete.
	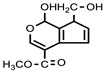 Genipin	Primary amines and aldehydes	Independent of pH	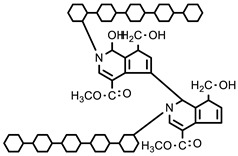	Non-toxic crosslinker and can undergo self-polymerization
	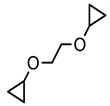 EGDMA	Primary amines and oxiranes	Basic pH and at high temperature	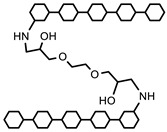	Weak base, reaction takes one hour. Hydrogels beads form.

**Table 2:** Crosslinking of polymers through reactive functional groups.

Reaction	Reaction Conditions	Reactive Polymer Groups	Crosslinkage	Comments
Schiff base mechanism	Neutral pH	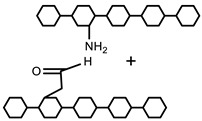	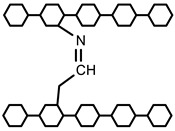	Good candidate for in situ gel formation. Reaction takes minimum 10 min.
Disulfide bonding	Neutral pH	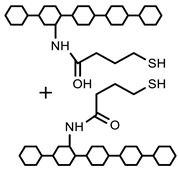	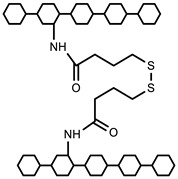	Good candidate for in situ gel formation and hydrogels have good mucoadhesivity.
Michael addition	Weak base and in presence of catalyst	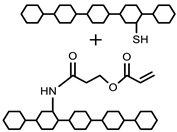	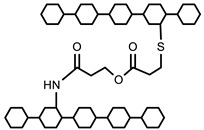	Good candidate for in situ gel formation and hydrogels have good mucoadhesivity.

**Table 3:** Crosslinking of polymers through small molecules and light-sensitive functional groups (Continued).

	Agent	Target Functional Group	Reaction Conditions	Crosslinkage	Comments
**Light sensitive groups**	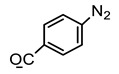 Functional azides	Primary amines	pH-Independent	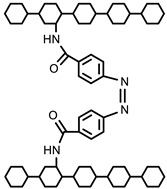	Multi step crosslinker. Suitable for injectable hydrogel synthesis.
	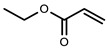 Functional acrylates	Other acrylic acids	pH-Independent	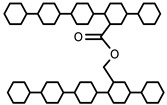	Multi step crosslinker. Suitable for injectable hydrogel synthesis.
